# White matter diffusion estimates in obsessive-compulsive disorder across 1653 individuals: machine learning findings from the ENIGMA OCD Working Group

**DOI:** 10.1038/s41380-023-02392-6

**Published:** 2024-02-07

**Authors:** Bo-Gyeom Kim, Gakyung Kim, Yoshinari Abe, Pino Alonso, Stephanie Ameis, Alan Anticevic, Paul D. Arnold, Srinivas Balachander, Nerisa Banaj, Nuria Bargalló, Marcelo C. Batistuzzo, Francesco Benedetti, Sara Bertolín, Jan Carl Beucke, Irene Bollettini, Silvia Brem, Brian P. Brennan, Jan K. Buitelaar, Rosa Calvo, Miguel Castelo-Branco, Yuqi Cheng, Ritu Bhusal Chhatkuli, Valentina Ciullo, Ana Coelho, Beatriz Couto, Sara Dallaspezia, Benjamin A. Ely, Sónia Ferreira, Martine Fontaine, Jean-Paul Fouche, Rachael Grazioplene, Patricia Gruner, Kristen Hagen, Bjarne Hansen, Gregory L. Hanna, Yoshiyuki Hirano, Marcelo Q. Höxter, Morgan Hough, Hao Hu, Chaim Huyser, Toshikazu Ikuta, Neda Jahanshad, Anthony James, Fern Jaspers-Fayer, Selina Kasprzak, Norbert Kathmann, Christian Kaufmann, Minah Kim, Kathrin Koch, Gerd Kvale, Jun Soo Kwon, Luisa Lazaro, Junhee Lee, Christine Lochner, Jin Lu, Daniela Rodriguez Manrique, Ignacio Martínez-Zalacaín, Yoshitada Masuda, Koji Matsumoto, Maria Paula Maziero, Jose M. Menchón, Luciano Minuzzi, Pedro Silva Moreira, Pedro Morgado, Janardhanan C. Narayanaswamy, Jin Narumoto, Ana E. Ortiz, Junko Ota, Jose C. Pariente, Chris Perriello, Maria Picó-Pérez, Christopher Pittenger, Sara Poletti, Eva Real, Y. C. Janardhan Reddy, Daan van Rooij, Yuki Sakai, João Ricardo Sato, Cinto Segalas, Roseli G. Shavitt, Zonglin Shen, Eiji Shimizu, Venkataram Shivakumar, Noam Soreni, Carles Soriano-Mas, Nuno Sousa, Mafalda Machado Sousa, Gianfranco Spalletta, Emily R. Stern, S. Evelyn Stewart, Philip R. Szeszko, Rajat Thomas, Sophia I. Thomopoulos, Daniela Vecchio, Ganesan Venkatasubramanian, Chris Vriend, Susanne Walitza, Zhen Wang, Anri Watanabe, Lidewij Wolters, Jian Xu, Kei Yamada, Je-Yeon Yun, Mojtaba Zarei, Qing Zhao, Xi Zhu, ENIGMA-OCD Working Group, Honami Arai, Ana Isabel Araújo, Kentaro Araki, Paul D. Arnold, Justin T. Baker, Núria Bargalló, Sara Bertolín, John R. Best, Premika S. W. Boedhoe, Sven Bölte, Vilde Brecke, Jan K. Buitelaar, Rosa Calvo, Carolina Cappi, Joao Castelhano, Wei Chen, Sutoh Chihiro, Kang Ik Kevin Cho, Sunah Choi, Daniel Costa, Nan Dai, Shareefa Dalvie, Damiaan Denys, Juliana B. Diniz, Isabel C. Duarte, Calesella Federico, Jamie D. Feusner, Kate D. Fitzgerald, Egill Axfjord Fridgeirsson, Edna Grünblatt, Sayo Hamatani, Gregory Hanna, Mengxin He, Odile A. van den Heuvel, Marcelo Q. Höxter, Morgan Hough, Keisuke Ikari, Jonathan Ipser, Hongyan Jiang, Linling Jiang, Niels T. de Joode, Norbert Kathmann, Taekwan Kim, Hitomi Kitagawa, Masaru Kuno, Yoo Bin Kwak, Jun Soo Kwon, Wieke van Leeuwen, Chiang-shan Ray Li, Na Li, Yanni Liu, Fang liu, Antonio Carlos Lopes, Jin Lu, Yuri Milaneschi, Hein van Marle, Sergi Mas, David Mataix-Cols, Maria Alice de Mathis, Maria Paula Mazieiro, Sarah Medland, Renata Melo, Euripedes C. Miguel, Astrid Morer, Alessandro S. De Nadai, Tomohiro Nakao, Masato Nihei, Luke Norman, Erika L. Nurmi, Joseph O’Neil, Sanghoon Oh, Sho Okawa, John C. Piacentini, Maria Picó-Pérez, Natalia Rodriguez, Daan van Rooij, João R. Sato, Cinto Segalas, Renata Silva, Noam Soreni, Michael Stevens, Anouk van der Straten, Jumpei Takahashi, Tais Tanamatis, Jinsong Tang, Anders Lillevik Thorsen, David Tolin, Anne Uhlmann, Benedetta Vai, Ysbrand D. van der Werf, Dick J. Veltman, Nora Vetter, Jicai Wang, Cees J. Weeland, Guido A. van Wingen, Stella J. de Wit, Nicole Wolff, Xiufeng Xu, Tokiko Yoshida, Fengrui Zhang, Paul M. Thompson, Willem B. Bruin, Guido A. van Wingen, Federica Piras, Fabrizio Piras, Dan J. Stein, Odile A. van den Heuvel, Helen Blair Simpson, Rachel Marsh, Jiook Cha

**Affiliations:** 1 Department of Psychology, College of Social Sciences, Seoul National University, Seoul, Republic of Korea; 2 Department of Brain and Cognitive Sciences, College of Natural Sciences, Seoul National University, Seoul, Republic of Korea; 3 Graduate School of Medical Science, Kyoto Prefectural University of Medicine, Department of Psychiatry, Kyoto City, Japan; 4 Bellvitge Biomedical Research Insitute-IDIBELL, Bellvitge University Hospital, Barcelona, Spain; 5 CIBER of Mental Health (CIBERSAM), Carlos III Health Institute, Madrid, Spain; 6 Department of Clinical Sciences, University of Barcelona, Barcelona, Spain; 7 The Margaret and Wallace McCain Centre for Child, Youth & Family Mental Health and Campbell Family Mental Health Research Institute, Centre for Addiction and Mental Health, Toronto, ON Canada; 8 Department of Psychiatry, University of Toronto, Toronto, ON Canada; 9 Program in Neurosciences and Mental Health, The Hospital for Sick Children, Toronto, ON Canada; 10 Department of Psychiatry, Yale University School of Medicine, New Haven, CT 06510 USA; 11 The Mathison Centre for Mental Health Research & Education, Hotchkiss Brain Institute, Cumming School of Medicine, University of Calgary, Calgary, AB Canada; 12 Departments of Psychiatry and Medical Genetics, Cumming School of Medicine, University of Calgary, Calgary, AB Canada; 13 OCD clinic, Department of Psychiatry, National Institute of Mental Health And Neurosciences (NIMHANS), Bangalore, India; 14 Laboratory of Neuropsychiatry, Department of Clinical and Neuroscience and Neurorehabilitation, IRCCS Santa Lucia Foundation, Rome, Italy; 15 Center of Image Diagnostic, Hospital Clínic de Barcelona, Barcelona, Spain; 16 Magnetic Resonance Image Core Facility, Institut d’Investigacions Biomèdiques August Pi i Sunyer (IDIBAPS), Barcelona, Spain; 17 Departamento e Instituto de Psiquiatria do Hospital das Clinicas, IPQ HCFMUSP, Faculdade de Medicina, Universidade de Sao Paulo, Sao Paulo, SP Brazil; 18 Department of Methods and Techniques in Psychology, Pontifical Catholic University, São Paulo, SP Brazil; 19 Vita-Salute San Raffaele University, Milano, Italy; 20 Psychiatry & Clinical Psychobiology, Division of Neuroscience, IRCCS Scientific Institute Ospedale San Raffaele, Milano, Italy; 21 Bellvitge Biomedical Research Institute-IDIBELL, Bellvitge University Hospital, Barcelona, Spain; 22 Department of Psychology, Humboldt-Universitat zu Berlin, Berlin, Germany; 23 Department of Clinical Neuroscience, Centre for Psychiatric Research and Education, Karolinska Institutet, Stockholm, Sweden; 24 Department of Medical Psychology, Medical School Hamburg, Hamburg, Germany; 25 Department of Child and Adolescent Psychiatry and Psychotherapy, University Hospital of Psychiatry Zurich, University of Zurich, Zurich, Switzerland; 26 Neuroscience Center Zurich, University of Zurich and ETH Zurich, Zurich, Switzerland; 27 McLean Hospital, Belmont, MA USA; 28 Department of Psychiatry, Harvard Medical School, Boston, MA USA; 29 Radboudumc, Department of Cognitive Neuroscience, Nijmegen, The Netherlands; 30 Karakter Child and Adolescent Psychiatry University Center, Nijmegen, The Netherlands; 31 Department of Child and Adolescent Psychiatry and Psychology, Institute of Neurosciences, Hospital Clínic Universitari, Barcelona, Spain; 32 Institut d’Investigacions Biomèdiques August Pi i Sunyer (IDIBAPS), Barcelona, Spain; 33 Department of Medicine, University of Barcelona, Barcelona, Spain; 34 Coimbra Institute for Biomedical Imaging and Translational Research (CIBIT), University of Coimbra, 3000-548 Coimbra, Portugal; 35 Institute for Nuclear Sciences Applied to Health (ICNAS), University of Coimbra, 3000-548 Coimbra, Portugal; 36 Faculty of Medicine, University of Coimbra, 3000-548 Coimbra, Portugal; 37 Department of Psychiatry, First Affiliated Hospital of Kunming Medical University, Kunming, China; 38 Research Center for Child Mental Development, Chiba University, Chiba, Japan; 39 United Graduate School of Child Development, Osaka University, Kanazawa University, Hamamatsu University, Chiba University and University of Fukui, Suita, Japan; 40 Life and Health Sciences Research Institute (ICVS), School of Medicine, University of Minho, Braga, Portugal; 41 ICVS/3B’s, PT Government Associate Laboratory, Braga/Guimaraes, Portugal; 42 Clinical Academic Center - Braga, Braga, Portugal; 43 Psychiatry & Clinical Psychobiology Unit, Division of Neuroscience, Scientific Institute Ospedale San Raffaele, Milano, Italy; 44 Department of Psychiatry and Behavioral Sciences, Albert Einstein College of Medicine, Bronx, NY USA; 45 Columbia University Medical College, Columbia University, New York, NY USA; 46 SAMRC Genomics of Brain Disorders Unit, Department of Psychiatry, Cape Town, South Africa; 47 Hospital of Molde, Møre og Romsdal Hospital Trust, Molde, Norway; 48 Bergen Center for Brain Plasticity, Haukeland University Hospital, Bergen, Norway; 49 Centre for Crisis Psychology, University of Bergen, Bergen, Norway; 50 Department of Psychiatry, University of Michigan Medical School, Ann Arbor, MI USA; 51 Highfield Unit Oxford, Warneford Hospital, Warneford Lane, Headington, Oxford, Oxfordshire OX3 7JX UK; 52 Shanghai Mental Health Center, Shanghai, China; 53 Levvel, academic center for child and adolescent care, Amsterdam, The Netherlands; 54 Department of Child and Adolescent Psychiatry, Amsterdam UMC, Amsterdam, The Netherlands; 55 Department of Communication Sciences and Disorders, University of Mississippi, Oxford, MS USA; 56 Imaging Genetics Center, Mark and Mary Stevens Neuroimaging and Informatics Institute, Keck School of Medicine, University of Southern California, Marina del Rey, Los Angeles, CA USA; 57 Department of Psychiatry University of Oxford, Warneford Hospital, Oxford, OX3 7JX UK; 58 Department of Psychiatry, University of British Columbia, Vancouver, BC Canada; 59 BC Children’s Hospital Research Institute, Vancouver, BC Canada; 60 Amsterdam UMC, Vrije Universteit Amsterdam, Department of Psychiatry, Amsterdam Neuroscience, Amsterdam, The Netherlands; 61 Amsterdam UMC, Vrije Universiteit Amsterdam, Department of Anatomy and Neurosciences, Amsterdam Neuroscience, Amsterdam, The Netherlands; 62 Department of Neuropsychiatry, Seoul National University Hospital, Seoul, Republic of Korea; 63 Department of Psychiatry, Seoul National University College of Medicine, Seoul, Republic of Korea; 64 TUM-Neuroimaging Center (TUM-NIC) of Klinikum rechts der Isar, Technische Universitat Munchen, München, Germany; 65 Department of Diagnostic and Interventional Neuroradiology, School of Medicine, Technical University of Munich, Munich, Germany; 66 Department of Clinical Psychology, University of Bergen, Bergen, Norway; 67 Department of Brain and Cognitive Sciences, Seoul National University College of Natural Sciences, Seoul, Republic of Korea; 68 Institute of Human Behavioral Medicine, SNU-MRC, Seoul, Republic of Korea; 69 Department of Psychiatry, Uijeongbu Eulji Medical Center, Uijeongbu, Republic of Korea; 70 SAMRC Unit on Risk & Resilience in Mental Disorders, Department of Psychiatry, Stellenbosch University, Stellenbosch, South Africa; 71 Department of Psychiatry, First Affiliated Hospitalof Kunming Medical University, Kunming, China; 72 Graduate School of Systemic Neurosciences, Ludwig-Maximilians-University, Munich, Germany; 73 Department of Radiology, Bellvitge University Hospital, Barcelona, Spain; 74 Chiba University Hospital, Chiba University, Chiba, Japan; 75 LIM 23, Instituto de Psiquiatria, Hospital das Clinicas da Faculdade de Medicina da Universidade de Sao Paulo, Sao Paulo, Brazil; 76 Faculty of Medicine, City University of Sao Paulo, Sao Paulo, Brazil; 77 Anxiety Treatment and Research Clinic, St. Joseph’s Hamilton Healthcare, Hamilton, ON Canada; 78 Dapartmente of Psychiatry and Behavioural Neurosciences, McMaster University, Hamilton, ON Canada; 79 Psychological Neuroscience Lab, CIPsi, School of Psychology, University of Minho, Braga, Portugal; 80 Department of Psychiatry, Graduate School of Medical Science, Kyoto Prefectural University of Medicine, Kyoto, Japan; 81 University of Illinois at Urbana-Champaign, Champaign, IL USA; 82 Departamento de Psicología Básica, Clínica y Psicobiología, Universitat Jaume I, Castelló de la Plana, Spain; 83 Department of Psychology, Yale University, New Haven, CT USA; 84 Child Study Center, Yale University, New Haven, CT USA; 85 Center for Brain and Mind Health, Yale University, New Haven, CT USA; 86 Radboud University Medical Center, Donders Institute for Brain, Cognition and Behavior, Department of Cognitive Neuroscience, Nijmegen, The Netherlands; 87 ATR Brain Information Communication Research Laboratory Group, Kyoto, Japan; 88 Center of Mathematics, Computing and Cognition, Universidade Federal do ABC, Santo André, Brazil; 89 Big Data, Hospital Israelita Albert Einstein, São Paulo, Brazil; 90 Departamento de Psiquiatria, Hospital das Clinicas HCFMUSP, Faculdade de Medicina, Universidade de Sao Paulo, Sao Paulo, SP Brazil; 91 Department of Cognitive Behavioral Physiology, Graduate School of Medicine, Chiba University, Chiba, Japan; 92 National Institute of Mental Health and Neurosciences, Department of Integrative Medicine, Bengaluru, India; 93 Department of Psychiatry and Behavioural Neurosciences, McMaster University, Hamilton, Ontario Canada; 94 Offord Centre for Child Studies, Hamilton, Ontario Canada; 95 Department of Social Psychology and Quantitative Psychology, University of Barcelona, Barcelona, Spain; 96 Division of Neuropsychiatry, Menninger Department of Psychiatry and Behavioral Science, Baylor College of Medicine, Houston, TX USA; 97 Department of Psychiatry, New York University School of Medicine, New York, NY USA; 98 Clinical Research, Nathan Kline Institute for Psychiatric Research, Orangeburg, NY USA; 99 British Columbia Children’s Hospital, Psychiatry, Vancouver, BC Canada; 100 British Columbia Mental Health and Substance Use Services Research Institute, Vancouver, BC Canada; 101 Departments of Psychiatry and Neuroscience, Icahn School of Medicine at Mount Sinai, New York, NY USA; 102 Mental Illness Research, Education and Clinical Center, James J. Peters VA Medical Center, Bronx, NY USA; 103 Weill-Cornell Medicine Qatar, Education City, Doha, Qatar; 104 Amsterdam Neuroscience, Compulsivity, Impulsivity & Attention program, Amsterdam, The Netherlands; 105 Shanghai Mental Health Center, Shanghai Jiao Tong University School of Medicine, Shanghai, China; 106 Norwegian University of Science and Technology (NTNU), Faculty of Medicine, Regional Centre for Child and Youth Mental Health and Child Welfare (RKBU Central Norway), Klostergata 46, 7030 Trondheim, Norway; 107 Department of Internal Medicine, First Affiliated Hospital of Kunming Medical University, Kunming, China; 108 Department of Radiology, Graduate School of Medical Science, Kyoto Prefectural University of Medicine, Kyoto, Japan; 109 Seoul National University Hospital, Seoul, Republic of Korea; 110 Yeongeon Student Support Center, Seoul National University College of Medicine, Seoul, Republic of Korea; 111 Institute of Medical Science and Technology, Shahid Beheshti University, Tehran, Iran; 112 Department of Psychiatry, Columbia University Irving Medical Center, New York, NY USA; 113 New York State Psychiatric Institute, New York, NY USA; 114 Amsterdam UMC, Universiteit van Amsterdam, Department of Psychiatry, Amsterdam, The Netherlands; 115 Department of Psychiatry and Mental Health, University of Cape Town, Cape Town, South Africa; 116 SAMRC Unit on Risk & Resilience in Mental Disorders, Cape Town, South Africa; 117 CIBERSAM, Barcelona, Spain; 118 Gerontology Research Centre, Simon Fraser University, Burnaby, BC Canada; 119 Amsterdam UMC, Vrije Universiteit Amsterdam, Department of Psychiatry, Department of Anatomy & Neurosciences, Amsterdam, The Netherlands; 120 Department of Women’s & Children’s Health, Center for Psychiatry Research, Karolinska Institutet, Stockholm, Sweden; 121 Magnetic Resonance Image Center, First Affiliated Hospital of Kunming Medical University, Kunming, China; 122 Department of Cognitive Behavioral Physiology, Graduate School of Medicine and School of Medicine, Chiba University, Chiba, Japan; 123 Psychiatry Neuroimaging Laboratory, Department of Psychiatry, Brigham and Women’s Hospital, Harvard Medical School, Boston, MA USA; 124 SA MRC Unit on Risk & Resilience in Mental Disorders, Department of Psychiatry and Mental Health, University of Cape Town, Cape Town, South Africa; 125 Amsterdam UMC, University of Amsterdam, Department of Psychiatry, Amsterdam Neuroscience, Amsterdam, The Netherlands; 126 Division of Neurosciences & Clinical Translation, Temerty Faculty of Medicine, University of Toronto, Toronto, ON Canada; 127 Department of Neuropsychiatry, Graduate School of Medical Sciences, Kyushu University, Fukuoka, Japan; 128 Department of Psychiatry and Mental Health and Neuroscience Institute, Brain Behaviour Unit, University of Cape Town, Cape Town, South Africa; 129 Department of Bio and Brain Engineering, Korea Advanced Institute of Science and Technology, Daejeon, Republic of Korea; 130 Amsterdam UMC, Vrije Universiteit Amsterdam, Department of Psychiatry, Amsterdam Neuroscience, Amsterdam, The Netherlands; 131 Amsterdam UMC, Vrije Universiteit, Department of Psychiatry, Amsterdam Neuroscience, Amsterdam, The Netherlands; 132 Department of Basic Clinical Practice, Pharmacology Unit, University of Barcelona, Barcelona, Spain; 133 IDIBAPS, Barcelona, Spain; 134 Centro de Investigación Biomédica en Red de salud mental (CIBERSAM), Barcelona, Spain; 135 QIMR Berghofer Medical Research Institute, Brisbane, QLD Australia; 136 Department of Child and Adolescent Psychiatry and Psychology, Hospital Clínic of Barcelona. CIBERSAM, Barcelona, Spain; 137 Texas State University, Austin, TX USA; 138 The National Institutes of Health (NIH), Bethesda, MD USA; 139 Department of Psychiatry, University of California, Los Angeles, CA USA; 140 Division of Child and Adolescent Psychiatry, Jane & Terry Semel Institute For Neurosciences, University of California, Los Angeles, CA USA; 141 Pediatric OCD Consultation Clinic, McMaster University, Hamilton, ON Canada; 142 Anxiety Treatment and Research Center, McMaster University, Hamilton, ON Canada; 143 Institute of Living, Hartford, CT USA; 144 Yale University School of Medicine, New Haven, CT USA; 145 Department of Psychiatry, Zhejiang University School of Medicine, Hangzhou, China; 146 Department of Child and Adolescent Psychiatry and Psychotherapy, TU Dresden, Dresden, Germany; 147 Department of Psychology, MSB Medical School Berlin, Berlin, Germany

**Keywords:** Neuroscience, Diagnostic markers

## Abstract

White matter pathways, typically studied with diffusion tensor imaging (DTI), have been implicated in the neurobiology of obsessive-compulsive disorder (OCD). However, due to limited sample sizes and the predominance of single-site studies, the generalizability of OCD classification based on diffusion white matter estimates remains unclear. Here, we tested classification accuracy using the largest OCD DTI dataset to date, involving 1336 adult participants (690 OCD patients and 646 healthy controls) and 317 pediatric participants (175 OCD patients and 142 healthy controls) from 18 international sites within the ENIGMA OCD Working Group. We used an automatic machine learning pipeline (with feature engineering and selection, and model optimization) and examined the cross-site generalizability of the OCD classification models using leave-one-site-out cross-validation. Our models showed low-to-moderate accuracy in classifying (1) “OCD vs. healthy controls” (Adults, receiver operator characteristic-area under the curve = 57.19 ± 3.47 in the replication set; Children, 59.8 ± 7.39), (2) “unmedicated OCD vs. healthy controls” (Adults, 62.67 ± 3.84; Children, 48.51 ± 10.14), and (3) “medicated OCD vs. unmedicated OCD” (Adults, 76.72 ± 3.97; Children, 72.45 ± 8.87). There was significant site variability in model performance (cross-validated ROC AUC ranges 51.6–79.1 in adults; 35.9–63.2 in children). Machine learning interpretation showed that diffusivity measures of the corpus callosum, internal capsule, and posterior thalamic radiation contributed to the classification of OCD from HC. The classification performance appeared greater than the model trained on grey matter morphometry in the prior ENIGMA OCD study (our study includes subsamples from the morphometry study). Taken together, this study points to the meaningful multivariate patterns of white matter features relevant to the neurobiology of OCD, but with low-to-moderate classification accuracy. The OCD classification performance may be constrained by site variability and medication effects on the white matter integrity, indicating room for improvement for future research.

## Introduction

Obsessive-compulsive disorder (OCD) is a common, often chronic psychiatric disorder, affecting 1.0–1.5% of the global population over their lifetime [1]. Extensive neuroimaging research suggests structural and functional abnormalities in cortico-striato-thalamo-cortical (CSTC) circuits in OCD [2–7]. The field has also started to address the question of whether multivariate analyses of neuroimaging data can be used to classify OCD [8, 9].

Prior OCD studies with relatively small to modest samples show mixed findings, with OCD classification accuracies varying from 66% to 100% [8]. However, the generalizability of such findings has rarely been tested, and reproducibility failures have been a major challenge in psychiatric neuroimaging [9–12]. Indeed, typical single-site neuroimaging studies seeking brain-wide associations with psychopathology using small sample sizes of tens to hundreds of individuals may report inflated effect sizes, decreasing reproducibility [13].

The ENIGMA-OCD consortium has allowed rigorous mega-analyses and meta-analyses based on the largest international multisite neuroimaging datasets to date [9]. A machine learning analysis of regional measures of cortical thickness, surface area and subcortical volume found that model performance did not exceed chance-level, but that classification performance was improved when individuals with OCD were grouped according to medication status.

Altered white matter pathways have been implicated in the neurobiology of OCD [14]. An ENIGMA-OCD study using diffusion tensor imaging reported significantly lower fractional anisotropy (FA) in the sagittal striatum (SS) and posterior thalamic radiation (PTR), higher mean diffusivity (MD) in the SS and higher radial diffusivity (RD) in SS and PTR [15]. However, the question of whether white matter diffusion tensor imaging findings can be used to classify OCD has not yet been explored in large and multisite studies.

In this study, we therefore used ENIGMA-OCD on diffusion tensor imaging to test the classification power of such measures in a large multisite sample of individuals with OCD and healthy controls. We tested several machine learning algorithms to distinguish those with OCD versus healthy controls, as well as to distinguish OCD individuals off medication versus healthy controls, and to distinguish OCD individuals on versus off medication. We also assessed the site-variability and reproducibility of predictive models using leave-one-site-out cross-validation and evaluated the utility of a post-processing harmonization tool (i.e., NeuroComBat). Finally, we employed a machine learning interpretation framework to assess which features were most relevant to the various classifications.

## Participants and methods

### Participants

Data from the ENIGMA-OCD Working Group recruited from 18 international research institutes were used. We analyzed data from 1653 participants, including 1336 adult participants (429 unmedicated OCD, 261 medicated OCD, 646 HC) and 317 pediatric participants (70 unmedicated OCD, 105 medicated OCD, 142 HC) (Table 1). Here, we defined pediatrics as under the age of 18 years old, consistent with previous work from the ENIGMA-OCD working group [2, 9]. The diagnosis of OCD and other comorbid conditions (i.e., anxiety disorders and major depressive disorder) were assessed using DSM-IV criteria (American Psychiatric Association, 2000). Clinical characteristics included medication status, childhood-onset, disease duration (in years), symptom severity (total scores ranging from 0-40 on the (Child) Yale-Brown Obsessive-Compulsive Scale ((C)Y-BOCS) [16, 17] and current or lifetime history of symptom dimensions (i.e., aggression/checking, cleaning/contamination, sexual/religion, hoarding, ordering/symmetry). Participants who did not have medication information were excluded from the medication classification analysis.

**Table 1:** Demographic and clinical characteristics of patients with obsessive-compulsive disorder (OCD) and healthy controls (HCs).

Characteristics	Adult OCD sample (*n* = 690)	Adult HC sample (*n* = 646)	Pediatric OCD sample (*n* = 175)	Pediatric HC sample (*n* = 142)
Demographic Characteristics				
Age (years)	31.6 ± 9.78	30.8 ± 9.97	14.5 ± 2.3	14.3 ± 2.4
Male *N* (%)	397 (25.6)	380 (24.2)	97 (27.8)	77 (22.1)
Clinical Characteristics				
OCD illness severity score	25 ± 7.11		20.8 ± 8.0	
Childhood-onset *N* (%)	351 (51.7)			
Duration of illness	12.4 ± 11.1		3.0 ± 2.5	
Medication use at time of scan *N* (%)	261 (37.8)		105 (60)	
Lifetime diagnosis				
Anxiety	76 (11.02)		48 (27.4)	
Major depression	84 (12.17)		18 (10.3)	
Current comorbid disorders				
Anxiety	69 (10.0)		29 (16.6)	
Major depression	77 (11.2)		6 (3.4)	
OCD symptom dimension				
Aggressive/checking	411 (59.6)		73 (41.7)	
Contamination/cleaning	355 (51.5)		62 (35.4)	
Symmetry/ordering	370 (53.6)		68 (38.9)	
Sexual/religious	228 (33.0)		55 (31.43)	
Hoarding	114 (16.5)		47 (26.9)	

Symptom score was indicated by total score on the adult and child version of the Yale-Brown Obsessive Compulsive Scales.

OCD symptom dimensions were measured with the YBOCS symptom checklist.

### Image acquisition and processing

Image preprocessing, including brain extraction, eddy current correction, movement correction, echo-planar imaging-induced distortion correction, and tensor fitting, was conducted at each site, and Tract-Based Spatial Statistics (TBSS) was performed using protocols and quality control pipelines provided by the ENIGMA-DTI working group (http://enigma.ini.usc.edu/protocols/dti-protocols/) [15]. For the entire skeleton in each hemisphere, four DTI measures (FA, MD, AD, and RD) were estimated within 25 tract-wise regions of interest (ROIs) based on the Johns Hopkins University (JHU) white matter parcellation atlas [15].

### OCD classification with machine learning

We conducted automated machine learning (AutoML) with H2O Driverless Artificial Intelligence (AI) (DAI, 1.8.7.1 version) using white matter anisotropy and diffusivity estimates (FA, MD, AD, RD; *N* = 252; 4 * {(19 fascicules * 3 (left, right, total) + 5 fascicules (total; e.g., corpus callosum, fornix) + average metrics across all fascicules)} and biological variables (age, sex). Three classification models were built in adult and pediatric samples, separately: (1) OCD vs. HC, (2) unmedicated OCD vs. HC (to test the effects of pure OCD–not confounded by medication effects–on the white matter), (3) medicated OCD vs. unmedicated OCD (to test the medication effects on the white matter). To prevent data leakage and reduce model overfitting, we split the entire data into a discovery set (80%) and a replication set (20%) (stratified by diagnosis). In the discovery set, we used leave-one-site-out (LOSO) cross-validation (11 sites for adults, seven sites for pediatrics) (Supplementary Fig. 1). With this scheme, within the discovery set, we evaluated the cross-site variability (or generalizability); within the replication set, we tested the overall model generalizability considering potential site variability. The test samples of the discovery data were not used during model optimization. The machine learning pipeline in AutoML involves the estimation of several base models (e.g., XGBoost, LightGBM, the general linear model (GLM)) and stacked ensemble models [18] derived from base models. The AutoML pipeline performs random hyperparameter tuning along with feature transformation (e.g., interaction encoding, numeric to categorical target encoding). Firstly, in each iteration, models learn and update the weights of the features and select important features based on the prior iteration. Then, the pipeline searches for the best feature transformations and model parameters using genetic algorithm [19]. In DAI, this procedure is called “feature evolution”. In genetic algorithm’s evolution can be seen as a competition between mutating parameters to find best “individuals” referring to information about feature transformations and hyperparameters. The feature evolution procedure is not entirely random and is informed from the variable importance interactions obtained from the modeling algorithms. So, this model training procedure including feature selection, transformation, and hyper-parameter tuning was performed using 11-fold-cross-validation scheme. In each fold, 10 folds were used for training the model, while the remaining 1-fold was used to (cross)validate the best training model. Finally, the best cross-validation models from each fold were combined and tested on a held-out replication set. In this way, the validation data within the 11-fold cross validation was not used for model optimization and feature evolution. Likely, replication data was not used for data preprocessing, model training or optimization. We used the ROC-AUC as the primary performance metric and accuracy, sensitivity, and specificity as additional metrics. pROC v. 1.16.2 in the R programming language was used to calculate the metrics [20].

### NeuroComBat harmonization

To reduce potential biases caused by site and scanner effects, we employed NeuroComBat harmonization [21]. ComBat, a short name for combatting batch effects when combining multiple batches [21, 22], corrects potential scanner/site effects on brain data by harmonizing the mean and variance of brain measures across scanners. We harmonized the diffusivity measures in the discovery and replication data separately while also including age and sex as covariates in the model matrix. Non-parametric empirical Bayes adjustments were used to adjust for batch effects.

### Model interpretation

To interpret the machine learning classifiers, we calculated the relative weights of DTI features contributing to OCD classification. We used two steps to determine the relative weights of DTI features contributing to OCD classification. First, we calculated the relative weights of each base model according to the model-specific algorithm. For LightGBM and XGBoostGBM, DAI computed the average reduction in impurity across all trees. Second, the importance of each base model was multiplied by its weight and normalized. We further implemented a machine learning interpretation framework, K-Local Interpretable Model-agnostic Explanation (K-LIME) [23]. This method fits surrogate linear models to data to extract the important features either positively or negatively associated with a target outcome: (1) OCD vs. HC, (2) unmedicated OCD vs. HC, and (3) medicated OCD vs. unmedicated OCD.

### Statistical analysis

To assess the effects of sites on diffusion white matter estimates, we performed principal component analysis (PCA). We tested the association between predicted OCD probabilities and clinical variables (e.g., medication status, childhood-onset) using stepwise regression models [24]. Additionally, we tested site effects on individual classification performances (i.e., whether participants were correctly classified as OCD or HC). To adjust for potential confounding factors, we included the following variables as covariates: age, sex, site, and average DTI metrics (i.e., mean FA, AD, RD, MD).

## Results

### Demographic characteristics

This study included 1336 adult participants (690 OCD, 646 HC) and 317 pediatric participants (175 OCD, 142 HC). Out of the adult OCD samples, 37.8% were taking medication, while 60% of the pediatric OCD sample were taking medication. OCD patients showed comorbidity with lifetime anxiety disorders (adult: 11.02%, pediatric: 27.4%) and major depressive disorder (adult: 12.2%, pediatric: 10.3%). Table 1 and Supplementary Table 1 contain detailed demographic and clinical characteristics of the participants. Demographic characteristics were not significantly different between OCD and HC (*P*’s > 0.45). However, the clinical characteristics varied across sites, including childhood-onset: ${X}^{2}$ = 93.66, *p* < 0.001, and symptom dimensions: Aggression/checking: ${X}^{2}$ = 64.33*, p* < 0.001, contamination/cleaning*:* ${X}^{2}$ = 53.02*, p* = 0.002, sexual/religious: ${X}^{2}$ = 46.33, *p* = 0.012, hoarding: ${X}^{2}$ = 73.06, *p* < 0.001, symmetry/ordering: ${X}^{2}$ = 145.03, *p* < 0.001 in adults. Illness duration also varied across sites in the pediatric samples, *F* = 13.20, *p* < 0.001.

### Classification of OCD

**Fig. 1:**
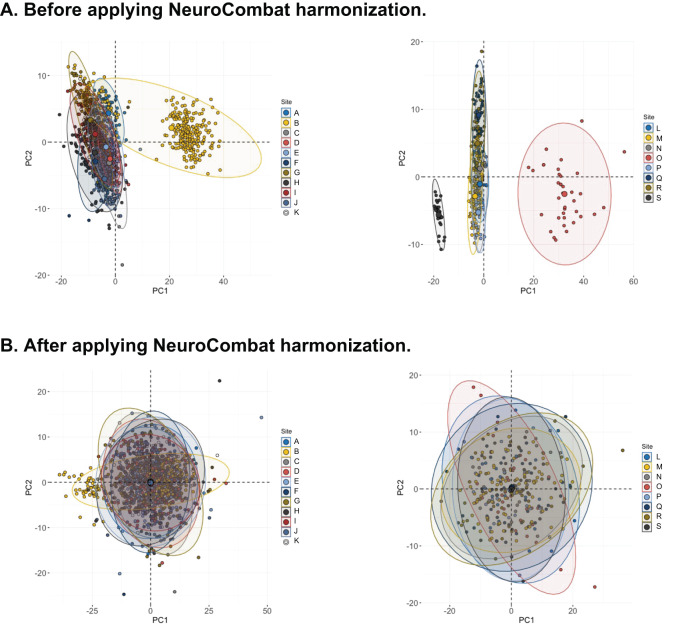
A biplot of principal component analysis (PCA) using the diffusion tensor estimates of the major white matter fascicules across the 18 international sites. **A** PCA biplot before applying NeuroCombat. (Left: Adult, Right: Pediatric). Some sites (e.g., site B) show apparent clusters distinct from the rest of the sites. **B** PCA biplot after applying NeuroCombat. (Left: Adult, Right: Pediatric).

**Fig. 2:**
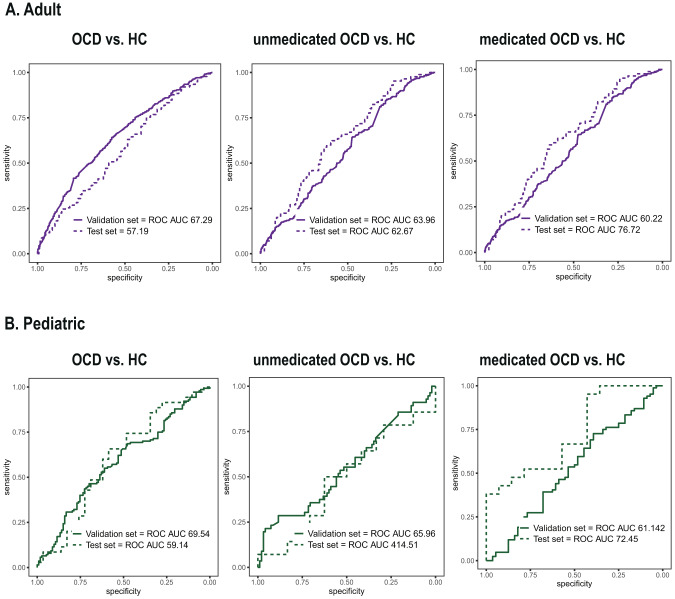
Classification of OCD diagnosis and medication status using diffusion tensor estimates. **A** Classification performances in adult samples. **B** Classification performances in pediatric samples.

The principal component analysis (PCA) of the four-diffusion metrics (FA, MD, AD, RD) across the 18 international sites revealed site variability (Fig. 1). In the PCA biplot, we observed two sites, one from adults and one from pediatrics, which were distinct from other sites. We then performed three classification tasks using the stacked ensemble machine learning models (LOSO cross-validation): (1) OCD vs. HC, (2) unmedicated OCD vs. HC, and (3) unmedicated OCD vs. medicated OCD (Tables 2 and 3, Fig. 2).

**Table 2:** Performance of classification of OCD clinical outcomes in (A) adult, (B) adult applied NeuroComBat harmonization, (C) pediatric, (D) pediatric applied NeuroCombat harmonization samples. ― mean with 95% confidence interval.

(A) Adult sample						
	OCD (*N* = 690) vs. HC (*N* = 646)		Unmedicated OCD (*N* = 429) vs. HC (*N* = 646)		Unmedicated OCD (*N* = 429) vs. medicated OCD (*N* = 261)	
	Discovery set	Replication set	Discovery set	Replication set	Discovery set	Replication set
ROC-AUC	67.29 ± 0.26	57.19 ± 3.47	63.96 ± 0.43	62.67 ± 3.84	60.22 ± 0.40	76.72 ± 3.97
Accuracy (%)	66.37 ± 0.27	57.08 ± 3.22	64.64 ± 0.49	61.68 ± 3.58	66.88 ± 0.32	67.15 ± 12.83
Sensitivity (%)	61.96 ± 0.79	75.36 ± 8.49	65.84 ± 1.66	58.82 ± 19.79	58.7 ± 1.53	92.31 ± 2.95
Specificity (%)	71.87 ± 0.73	37.69 ± 29.44	68.44 ± 1.00	63.57 ± 7.92	73.77 ± 1.44	51.76 ± 16.19

(B) Adult sample, NeuroComBat						
	OCD (*N* = 690) vs. HC (*N* = 646)		Unmedicated OCD (*N* = 429) vs. HC (*N* = 646)		Unmedicated OCD (*N* = 429) vs. medicated OCD (*N* = 261)	
	Discovery set	Replication set	Discovery set	Replication set	Discovery set	Replication set
ROC-AUC	64 ± 0.05	51.07 ± 3.54	67.35 ± 0.52	52.8 ± 4.18	66.12 ± 3.63	62.24 ± 5.08
Accuracy (%)	63.87 ± 0.07	53.36 ± 3.64	66.44 ± 0.52	60.75 ± 3.09	74.42 ± 0.65	68.6 ± 3.72
Sensitivity (%)	67.14 ± 1.20	37.68 ± 25.16	63.95 ± 1.80	37.65 ± 17.42	76.14 ± 0.71	48.08 ± 13.79
Specificity (%)	60.96 ± 1.24	70 ± 13.74	71.55 ± 0.87	75.97 ± 9.51	70.31 ± 1.35	81.18 ± 4.19

(C) Pediatric sample						
	OCD (*N* = 175) vs. HC (*N* = 142)		Unmedicated OCD (*N* = 105) vs. HC (*N* = 142)		Unmedicated OCD (*N* = 105) vs. medicated OCD (*N* = 70)	
	Discovery set	Replication set	Discovery set	Replication set	Discovery set	Replication set
ROC-AUC	69.54 ± 8.59	59.8 ± 7.39	65.96 ± 12.33	48.51 ± 10.14	61.82 ± 15.50	72.45 ± 8.87
Accuracy (%)	73.56 ± 6.82	62.5 ± 6.38	69.15 ± 8.35	57.9 ± 8.06	69.15 ± 11.18	74.3 ± 5.83
Sensitivity (%)	73.25 ± 17.25	65.71 ± 16.03	73.43 ± 14.12	50 ± 25.51	73.43 ± 12.74	95.24 ± 2.43
Specificity (%)	73.03 ± 13.18	58.62 ± 20.58	68.75 ± 9.90	62.5 ± 19.13	68.75 ± 15.95	42.86 ± 29.15

(D) Pediatric sample, NeuroComBat						
	OCD (*N* = 175) vs. HC (*N* = 142)		Unmedicated OCD (*N* = 105) vs. HC (*N* = 142)		Unmedicated OCD (*N* = 105) vs. medicated OCD (*N* = 70)	
	Discovery set	Replication set	Discovery set	Replication set	Discovery set	Replication set
ROC-AUC	66.05 ± 0.41	60.49 ± 7.20	60.71 ± 0.92	55.36 ± 10.15	66.78 ± 0.35	58.2 ± 8.85
Accuracy (%)	67.56 ± 0.38	62.5 ± 6.38	61.46 ± 0.28	71.05 ± 8.06	72.1 ± 0.28	60 ± 5.82
Sensitivity (%)	62.28 ± 1.55	71.43 ± 13.10	84.06 ± 0.91	35.71 ± 25.51	77.5 ± 0.91	47.61 ± 2.43
Specificity (%)	77.16 ± 1.46	51.72 ± 24.63	54.69 ± 2.05	91.67 ± 19.13	68.75 ± 2.05	78.57 ± 29.15

For the classification of medication status among OCD patients, some sites (i.e., Amsterdam, Shanghai) containing only unmedicated OCD were excluded from the discovery set.

For the classification of medication status among OCD patients, some sites (i.e., Calgary) containing only unmedicated OCD were excluded from the discovery set.

**Table 3:** The association between brain-predicted OCD risk probabilities and clinical features in a discovery set (stepwise regression).

Variable	Beta	F	*P* value	${\eta }^{2}$
**(A) Adult sample, Discovery set (OCD** = **379) (Adjusted** = **15.15%)**				
Site		6.996	7.72E−08	0.118
Age	0.011	16.152	7.10E−05	0.042
Hoarding	0.017	8.316	0.004	0.022
Childhood-onset	−0.010	4.172	0.042	0.011
Current Depression	0.015	2.372	0.124	0.006
**(B) Pediatric sample, Discovery set (OCD** = **55) (Adjusted** = **32.89%)**				
Site		11.796	6.57E−05	0.325
Depression	−0.13142	5.062	0.029	0.094
Aggression, Checking	−0.0645	4.619	0.037	0.086
Age	0.02355	1.896	0.175	0.037

In adult samples, the models minimally-to-modestly classified participants with OCD diagnosis from healthy controls in the discovery set (*N* = 1068, ROC AUC = 67.29 ± 0.26) and the replication set (*N* = 268, ROC AUC = 57.19 ± 3.47). The models also minimally-to-modestly distinguished unmedicated OCD versus healthy individuals in the discovery set (*N* = 854, ROC AUC = 63.96 ± 0.43) and the replication set (*N* = 214, ROC AUC = 62.67 ± 3.84). Finally, the models distinguished medicated OCD versus unmedicated OCD participants in the discovery set (*N* = 437, ROC AUC = 60.22 ± 0.40) and the replication set (*N* = 137, ROC AUC = 76.72 ± 3.97).

In pediatric samples, the models classified participants with OCD diagnosis versus healthy controls in the discovery set (*N* = 270, ROC AUC = 69.54 ± 8.59) and the replication set (*N* = 64, ROC AUC = 59.80 ± 7.39). The models also classified unmedicated OCD versus healthy individuals in the discovery set (*N* = 151, ROC AUC = 65.96 ± 12.33) and the replication set (*N* = 38, ROC AUC = 48.51 ± 10.14). Finally, the models classified medicated OCD versus unmedicated OCD participants in the discovery set (*N* = 140, ROC AUC = 61.82 ± 15.50) and the replication set (*N* = 35, ROC AUC = 72.45 ± 8.87) (Table 2C).

**Fig. 3:**
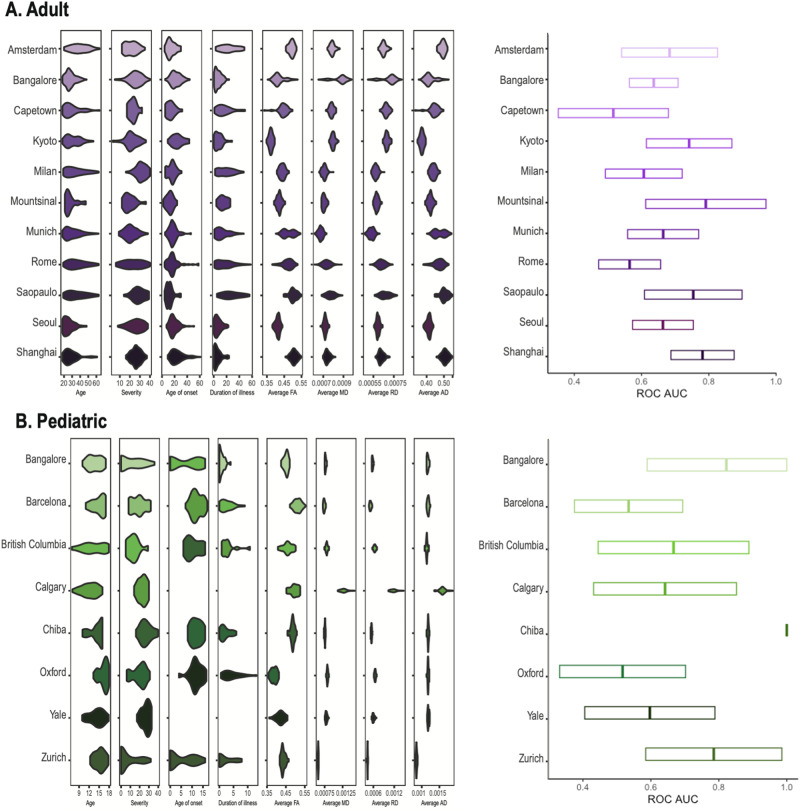
Sample characteristics and prediction performance (ROC AUC) across sites. **A** In adult samples. **B** In pediatric samples. Left: Violin plots of sociodemographic, clinical, and 763 brain features across sites, Right: Box plot of the area under the receiver operating 764 characteristic curve (ROC AUC) for the leave-one-site-out (LOSO) cross validation in the 765 diagnosis classification task (OCD vs. HC).

In classifying OCD and HC, the ROC AUC of adult samples ranged from 51.6% (site C) to 79.1% (site F), and pediatric samples ranged from 35.9% (site M) to 63.2% (site L) across sites. Also, mean values of DTI metrics across all ROIs showed significant differences across sites (*F*s > 97.4, *p* < 0.001). The site variability was significantly associated with the classification performance in OCD patients (χ^2^ = 57.19, *p* < 0.001) and HCs (χ^2^ = 50.30, *p* < 0.001) when adjusting for the covariates (Fig. 3).

### Classification of OCD with NeuroCombat-harmonized data

Considering the site variability (Fig. 1), we implemented the ML analysis with NeuroCombat-harmonized data to correct site effects. The NeuroComBat-harmonized data showed slightly lower performance in the adult samples (Table 2A) and slightly higher performance in the pediatric samples (Table 2B).

### Variables associated with OCD classification

Results of stepwise regression analysis indicated that, in adults, site (e.g., site H, site I), higher age, hoarding symptoms, and adult-onset were significantly associated with estimated OCD probabilities (*t* > 2.04, *p* < 0.05) (Table 3). In pediatric samples, site (e.g., site M, site S), lifetime diagnosis of depression, and aggression/checking symptoms significantly correlated with predicted OCD probabilities (*t* > 2.15, *p* < 0.05).

### Machine learning interpretation

**Fig. 4:**
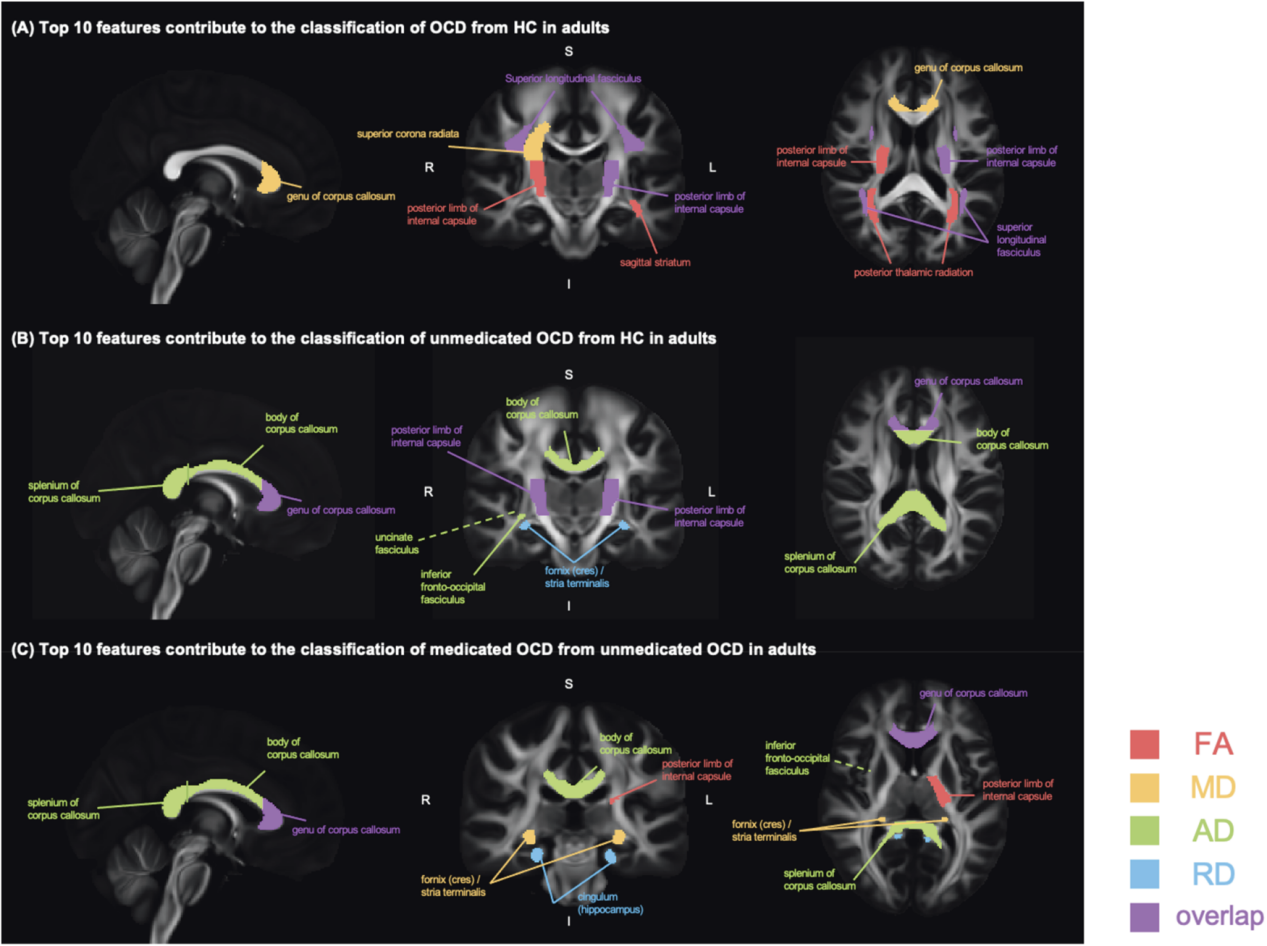
Top 10 features of classification models in adults. **A** Top 10 features contribute to the classification of OCD from HC in adults. **B** Top 10 features contribute to the classification of unmedicated OCD from HC in adults. **C** Top 10 features contribute to the classification of medicated OCD from unmedicated OCD in adults. Note: The color legend represents DTI measures: red for FA, yellow for MD, green for AD, and blue for RD. Regions with multiple DTI measures are highlighted in purple.

**Fig. 5:**
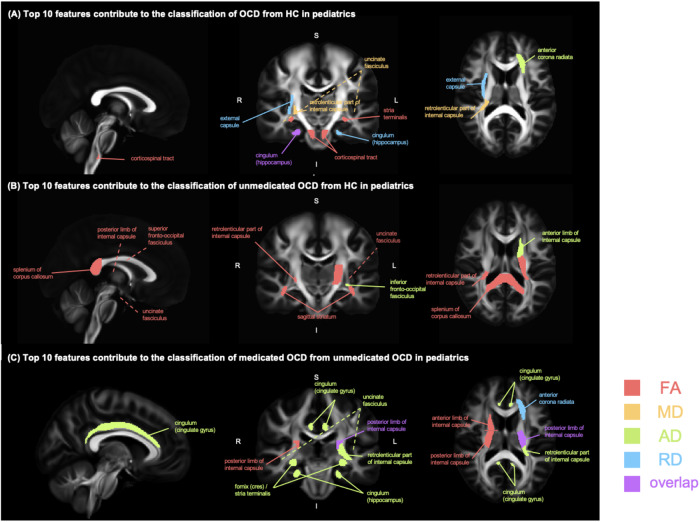
Top 10 features of classification models in pediatrics. **A** Top 10 features contribute to the classification of OCD from HC in pediatrics. **B** Top 10 features contribute to the classification of unmedicated OCD from HC in pediatrics. **C** Top 10 features contribute to the classification of medicated OCD from unmedicated OCD in pediatrics. Note: The color legend represents DTI measures: red for FA, yellow for MD, green for AD, and blue for RD. Regions with multiple DTI measures are highlighted in purple.

Our machine learning interpretation models showed that various specific diffusion white matter features contributed to the OCD classification (Figs. 4 and 5, Supplementary Fig. 2). For the classification of OCD from HC in adult samples, the top 10 features included the superior *corona radiata* (MD), age, posterior thalamic radiation (FA), and posterior limb of the internal capsule (FA, AD). In the pediatric samples, the cingulum (MD, AD), uncinate fasciculus (MD), fornix (FA), corticospinal tract (FA), and anterior *corona radiata* (AD) were important in classifying OCD diagnosis (Supplementary Fig. 2). In classifying unmedicated OCD and HC, the internal capsule contributed to both adult (FA, AD of posterior limb) and pediatric samples (FA of the retrolenticular part, AD of anterior limb, FA of posterior limb) (Fig. 4). In classifying medicated OCD and unmedicated OCD in adult samples, the top 10 features included the corpus callosum (total, genu), average FA, and average RD (Supplementary Fig. 2). For the pediatric samples, fornix and stria terminalis, cingulum (cingulate gyrus, hippocampus) were included in the top 10 features (Supplementary Fig. 2).

## Discussion

In this study, we tested the extent to the accuracy of machine learning in classifying the diagnosis or medication status of OCD patients based on white matter diffusion estimates obtained using the ENIGMA-matched image analysis pipeline across 18 international sites. Our results showed a low-to-moderate accuracy in predicting OCD diagnosis and medication status. Classification of medicated OCD versus unmedicated OCD had the best classification accuracy (ROC-AUC of 76.72 in adults), followed by unmediated OCD-health control classification (ROC-AUC of 63.96 in adults) and all OCD-HC (ROC-AUC of 57.19 in adults). In all OCD-HC classifications, the performance varied significantly across sites with cross-validated ROC AUC ranging 51.6–79.1 in adults, and 35.9–63.2 in children. Diffusion white matter features contributing to OCD classification (compared with HC) include anisotropy and diffusivity estimates of white matter in the internal capsule, thalamic radiation, and uncinate fasciculus.

The low-to-moderate accuracy of our machine learning models is consistent with prior work. OCD machine learning studies using structural MRI have found that accuracy in classifying OCD and HC, ranges from 60 to 90%, all in small datasets (*N* < 150) [8, 10]. However, these classification performances from small studies are likely to be inflated and not generalizable, while the true effect size (i.e., the brain-psychopathology association, regardless of the choice of analysis) may be smaller [13]. Indeed, a recent large-scale ENIGMA OCD study found that machine learning models trained on gray matter morphometric estimates from structural MRI resulted in poor classification of OCD vs. HC (ROC AUC, 51-54; leave-one-site CV) [9]. Our model based on white matter features showed improved classification performance compared with the gray matter morphometry model in adults and pediatric samples, though a direct comparison may not be warranted due to different machine learning pipelines and different subsamples used in this study. Future studies should determine whether multi-modal machine learning using structural and functional MRI can increase classification accuracy [25–28].

We observed significant site variability in classification performance. Firstly, this may be related to the variability of the quality of the diffusion MRI across sites. The aggregated ENIGMA MRI data were harmonized for the post-imaging processing procedure (e.g., TBSS) but not for data acquisition. Though this harmonization method was a best practice when the raw image data were not sharable, nevertheless, given the sensitivity of diffusion MRI to the image acquisition conditions (e.g., magnets types, pulse sequences, such as numbers of gradient directions or b values, etc.; compared with the gray matter morphometry validated across scanners, sites, and pulse sequence designs [29]), our approach is limited in controlling potential confounding factors and their impact on the quality of the diffusion white matter metrics. Also, our application of another post-processing harmonization method, NeuroComBat, was effective in matching the distributions of the data across the sites (in our PCA results). However, this method failed to result in a performance gain in the OCD classification (slightly higher AUC in pediatric samples, slightly lower AUC in adult samples) or a reduction of the cross-site variability. The covariate modeling with NeuroComBat also did not demonstrate a gain in performance. Secondly, our international multisite clinical samples show variability in clinical characteristics such as symptom severity, age, adult-onset, and duration of illness. The sampling variability may have added complexity to the already challenging task of OCD classification.

Our analysis of the machine learning model indicated that OCD probability was significantly associated with several sociodemographic and clinical characteristics. In adults with OCD, a higher age, adult onset, greater hoarding symptoms, and greater depressive symptoms were more likely to be predicted as having OCD. The significant correlation of age and adult-onset with the OCD likelihood might reflect age-dependent patterns in the diffusion white matter estimates. Though there are no significant group differences in age between OCD and HC, the neurobiology of OCD might be related to abnormal aging effects on the diffusion white matter estimates. Indeed, some literature shows that psychiatric disorders, including OCD and anxiety disorders, are linked to accelerated brain aging [30, 31]. However, the potential association between the neuropathophysiology of OCD and age appears more relevant to adults than to children because, despite the similar effect sizes of age and the OCD likelihood, only adult samples show statistical significance (probably due to a larger sample size). This may reflect the effects of chronicity in adult samples [32].

Our machine learning interpretation is consistent with prior white matter studies that have relied on univariate analyses and/or small sample sizes [33]. For example, the well-known CTSC pathway includes the internal capsule (posterior limb (FA, AD) in adults and retrolenticular part (MD) in children), which has been implicated in habit formation and cognitive control in OCD [34]. In the classification model of unmedicated OCD and HC, the corpus callosum - connecting the two cerebral hemispheres - was important in adults and pediatric samples alike. This finding is in line with the previous ENIGMA-OCD study [15] indicating that adult OCD was characterized by lower volume in the genu of the corpus callosum than HC. However, careful interpretation is needed because of differences in the brain metrics used, here based on tensor modeling (FA, MD). In addition, we found that the cingulum bundle contributed to the classification of unmedicated OCD and medicated OCD in both adult and pediatric samples. The cingulum bundle contains short and long connections between the frontal lobe, parietal lobe, and temporal lobe. In short, our machine learning findings suggest common patterns of white matter abnormalities in adult and pediatric OCD, as well as distinct patterns consistent with prior work [2].

The classification model of unmedicated OCD from HC showed greater accuracies than the model classifying all OCD from HC. This would suggest medication status likely confounds the white matter microstructure of OCD patients. In the literature, the causal effects of medication, Serotonin Reuptake Inhibitor (SSRI), on the white matter microstructure remain unclear: No randomized controlled trial exists. Nevertheless, given the key role of serotonin in neurodevelopment including gliogenesis [35], changes in extracellular serotonin levels in the brain owing to SSRI may impact the integrity of the white matter fibers. Prior correlational research supports this. A cross-sectional study shows a decrease in FA in the sagittal striatum associated with medication use in adults with OCD compared to unmedicated OCD [15]; longitudinal clinical studies show a decrease in MD of the midbrain white matter bundles after 12-week administration of SSRI [36], a decrease in MD in the frontal regions and the corpus callosum [37]. Though some of these correlational findings might indicate causal effects of SSRI on the white matter, nevertheless, without direct causal evidence it is still unclear if the associations result from the neurobiological effects of SSRI, symptom improvement, or both. A practical implication of our finding is that the diffusion white matter-based model presents a particular utility in classifying medication naïve individuals with OCD from healthy individuals. Though not reaching the clinical utility yet (e.g., around AUC of 80%), with further research (perhaps with the integration of brain, genetic, and behavioral multi-modal data [38]), the white matter diffusion estimates might be used to predict the risk for OCD. Future research may test whether the models trained on medication naïve OCD patients—perhaps capable of learning the neurobiological patterns underlying the OCD without medication confounding—may be used for related tasks (e.g., via representational learning [39].

There are limitations of this study. Firstly, the imaging acquisition was not harmonized across the sites, so we could not test whether the suboptimal model performance or the cross-site variability might result from the issues of the data or not. Given the sensitivity of the anisotropy and diffusivity estimates depending on the pulse sequence designs (e.g., the number of directions, b-values) [40], despite the harmonized image processing method (TBSS), the remaining data quality and validity issues perhaps may have worked against model performance. Secondly, since only the image-derived phenotypes were available from the ENIGMA consortium, but not the raw images, our results are only limited to a single type of analysis (TBSS) and metrics (diffusivity and anisotropy). Thirdly, our adult samples were larger than the pediatric samples, so our machine learning methods may have resulted in more optimized learning outcomes for adult samples.

In conclusion, using the largest multisite DTI with harmonized image processing, our investigation indicates that machine learning models currently allow only poor-to-modest classification power, but that capture meaningful multivariate patterns of white matter features relevant to the neurobiology of OCD. Accuracy is largely constrained by site variability, indicating room for future improvement.

### Supplementary information

Supplementral material

## Data Availability

The data that support the findings of this study are not openly available due to reasons of sensitivity and are available from the ENIGMA consortium (https://enigma.ini.usc.edu/) upon reasonable request.

## Code availability

The computer code for the above-described analyses is publicly available (https://github.com/Transconnectome/ENIGMA-OCD).

## References

[1] Fawcett EJ, Power H, Fawcett JM (2020). Women Are at Greater Risk of OCD Than Men. J Clin Psychiatry.

[2] Boedhoe PSW, Schmaal L, Abe Y, Ameis SH, Arnold PD, Batistuzzo MC (2017). Distinct Subcortical Volume Alterations in Pediatric and Adult OCD: A Worldwide Meta- and Mega-Analysis. Am J Psychiatry.

[3] de Wit SJ, Alonso P, Schweren L, Mataix-Cols D, Lochner C, Menchón JM (2014). Multicenter Voxel-Based Morphometry Mega-Analysis of Structural Brain Scans in Obsessive-Compulsive Disorder. Am J Psychiatry.

[4] Norman LJ, Carlisi C, Lukito S, Hart H, Mataix-Cols D, Radua J (2016). Structural and Functional Brain Abnormalities in Attention-Deficit/Hyperactivity Disorder and Obsessive-Compulsive Disorder. JAMA Psychiatry.

[5] Stein DJ, Costa DLC, Lochner C, Miguel EC, Reddy YCJ, Shavitt RG (2019). Obsessive–compulsive disorder. Nat Rev Dis Prim.

[6] Chamberlain SR, Menzies L, Hampshire A, Suckling J, Fineberg NA, del Campo N (2008). Orbitofrontal Dysfunction in Patients with Obsessive-Compulsive Disorder and Their Unaffected Relatives. Science.

[7] Menzies L, Chamberlain SR, Laird AR, Thelen SM, Sahakian BJ, Bullmore ET (2008). Integrating evidence from neuroimaging and neuropsychological studies of obsessive-compulsive disorder: The orbitofronto-striatal model revisited. Neurosci Biobehav Rev.

[8] Bruin W, Denys D, van Wingen G (2019). Diagnostic neuroimaging markers of obsessive-compulsive disorder: Initial evidence from structural and functional MRI studies. Prog Neuro Psychopharmacol Biol Psychiatry.

[9] Bruin W, Taylor L, Thomas RM, Shock JP, Zhutovsky P, Abe Y (2020). Structural neuroimaging biomarkers for obsessive-compulsive disorder in the ENIGMA-OCD consortium: medication matters. Transl Psychiatry.

[10] Zhou C, Cheng Y, Ping L, Xu J, Shen Z, Jiang L (2018). Support Vector Machine Classification of Obsessive-Compulsive Disorder Based on Whole-Brain Volumetry and Diffusion Tensor Imaging. Front Psychiatry.

[11] Yun J-Y, Jang JH, Kim SN, Jung WH, Kwon JS (2015). Neural Correlates of Response to Pharmacotherapy in Obsessive-Compulsive Disorder: Individualized Cortical Morphology-Based Structural Covariance. Prog Neuro Psychopharmacol Biol Psychiatry.

[12] Hoexter MQ, Miguel EC, Diniz JB, Shavitt RG, Busatto GF, Sato JR (2013). Predicting obsessive–compulsive disorder severity combining neuroimaging and machine learning methods. J Affect Disord.

[13] Marek S, Tervo-Clemmens B, Calabro FJ, Montez DF, Kay BP, Hatoum AS (2022). Reproducible brain-wide association studies require thousands of individuals. Nature.

[14] Radua J, Grau M, van den Heuvel O, Thiebaut de Schotten M, Stein D, Canales-Rodríguez E (2014). Multimodal Voxel-Based Meta-Analysis of White Matter Abnormalities in Obsessive–Compulsive Disorder. Neuropsychopharmacology.

[15] Piras F, Piras F, Abe Y, Agarwal SM, Anticevic A, Ameis S (2021). White matter microstructure and its relation to clinical features of obsessive–compulsive disorder: findings from the ENIGMA OCD Working Group. Translational. Psychiatry.

[16] Goodman WK, Price LH, Rasmussen SA, Mazure C, Fleischmann RL, Hill CL (1989). The Yale-Brown Obsessive Compulsive Scale. I. Development, use, and reliability. Arch Gen Psychiatry.

[17] Scahill L, Riddle MA, McSwiggin-Hardin M, Ort SI, King RA, Goodman WK (1997). Children’s Yale-Brown Obsessive Compulsive Scale: Reliability and Validity. J Am Acad Child Adolesc Psychiatry.

[18] van der Laan MJ, Polley EC, Hubbard AE (2007). Super Learner. Stat Appl Genet Mol Biol.

[19] Whitley D (1994). A genetic algorithm tutorial. Stat Comput.

[20] Robin X, Turck N, Hainard A, Tiberti N, Lisacek F, Sanchez J-C (2011). pROC: an open-source package for R and S+ to analyze and compare ROC curves. BMC Bioinforma.

[21] Fortin J-P, Parker D, Tunç B, Watanabe T, Elliott MA, Ruparel K (2017). Harmonization of multi-site diffusion tensor imaging data. NeuroImage.

[22] Fortin J-P, Cullen N, Sheline YI, Taylor WD, Aselcioglu I, Cook PA (2018). Harmonization of cortical thickness measurements across scanners and sites. NeuroImage.

[23] Ribeiro MT, Singh S, Guestrin C. Why Should I Trust You? In: Proceedings of the 22nd ACM SIGKDD International Conference on Knowledge Discovery and Data Mining - KDD ’16. 2016. 10.1145/2939672.2939778.

[24] Ganesh A, Ospel JM, Menon BK, Demchuk AM, McTaggart RA, Nogueira RG (2021). Assessment of Discrepancies Between Follow-up Infarct Volume and 90-Day Outcomes Among Patients With Ischemic Stroke Who Received Endovascular Therapy. JAMA Netw Open.

[25] Calhoun VD, Sui J (2016). Multimodal Fusion of Brain Imaging Data: A Key to Finding the Missing Link(s) in Complex Mental Illness. Biol Psychiatry Cogn Neurosci Neuroimaging.

[26] Kuo C-Y, Tai T-M, Lee P-L, Tseng C-W, Chen C-Y, Chen L-K (2021). Improving Individual Brain Age Prediction Using an Ensemble Deep Learning Framework. Front Psychiatry.

[27] Guggenmos M, Schmack K, Veer IM, Lett T, Sekutowicz M, Sebold M (2020). A multimodal neuroimaging classifier for alcohol dependence. Sci Rep.

[28] Menon SS, Krishnamurthy K (2021). Multimodal Ensemble Deep Learning to Predict Disruptive Behavior Disorders in Children. Front Neuroinformatics.

[29] Guo C, Ferreira D, Fink K, Westman E, Granberg T (2019). Repeatability and reproducibility of FreeSurfer, FSL-SIENAX and SPM brain volumetric measurements and the effect of lesion filling in multiple sclerosis. Eur Radiol.

[30] Liu L, Liu J, Yang L, Wen B, Zhang X, Cheng J (2022). Accelerated Brain Aging in Patients With Obsessive-Compulsive Disorder. Front Psychiatry.

[31] Han LKM, Schnack HG, Brouwer RM, Veltman DJ, van der Wee NJA, van Tol M-J (2021). Contributing factors to advanced brain aging in depression and anxiety disorders. Transl Psychiatry.

[32] Koch K, Reeß TJ, Rus OG, Zimmer C, Zaudig M (2014). Diffusion tensor imaging (DTI) studies in patients with obsessive-compulsive disorder (OCD): A review. J Psychiatr Res.

[33] Simpson HB, van den Heuvel OA, Miguel EC, Reddy YCJ, Stein DJ, Lewis-Fernández R (2020). Toward identifying reproducible brain signatures of obsessive-compulsive profiles: rationale and methods for a new global initiative. BMC Psychiatry.

[34] Spalletta G, Piras F, Fagioli S, Caltagirone C, Piras F (2014). Brain microstructural changes and cognitive correlates in patients with pure obsessive compulsive disorder. Brain Behav.

[35] Millard SJ, Weston-Green K, Newell KA (2017). The effects of maternal antidepressant use on offspring behaviour and brain development: Implications for risk of neurodevelopmental disorders. Neurosci Biobehav Rev.

[36] Fan Q, Yan X, Wang J, Chen Y, Wang X, Li C (2012). Abnormalities of White Matter Microstructure in Unmedicated Obsessive-Compulsive Disorder and Changes after Medication. PLoS ONE.

[37] Seiger R, Gryglewski G, Klöbl M, Kautzky A, Godbersen GM, Rischka L (2021). The Influence of Acute SSRI Administration on White Matter Microstructure in Patients Suffering From Major Depressive Disorder and Healthy Controls. Int J Neuropsychopharmacol.

[38] Rahaman MA, Chen J, Fu Z, Lewis N, Iraji A, Calhoun VD Multi-modal deep learning of functional and structural neuroimaging and genomic data to predict mental illness. IEEE Xplore. 2021:3267-72. https://ieeexplore.ieee.org/abstract/document/9630693. Accessed 25 July 2022. 10.1109/EMBC46164.2021.9630693 34891938

[39] Abrol A, Fu Z, Salman M, Silva R, Du Y, Plis S (2021). Deep learning encodes robust discriminative neuroimaging representations to outperform standard machine learning. Nat Commun.

[40] Ni H, Kavcic V, Zhu T, Ekholm S, Zhong J (2006). Effects of number of diffusion gradient directions on derived diffusion tensor imaging indices in human brain. AJNR Am J Neuroradiol.

