# Correction: White matter diffusion estimates in obsessive-compulsive disorder across 1653 individuals: machine learning findings from the ENIGMA OCD Working Group

**DOI:** 10.1038/s41380-024-02494-9

**Published:** 2024-03-07

**Authors:** Bo-Gyeom Kim, Gakyung Kim, Yoshinari Abe, Pino Alonso, Stephanie Ameis, Alan Anticevic, Paul D. Arnold, Srinivas Balachander, Nerisa Banaj, Nuria Bargalló, Marcelo C. Batistuzzo, Francesco Benedetti, Sara Bertolín, Jan Carl Beucke, Irene Bollettini, Silvia Brem, Brian P. Brennan, Jan K. Buitelaar, Rosa Calvo, Miguel Castelo-Branco, Yuqi Cheng, Ritu Bhusal Chhatkuli, Valentina Ciullo, Ana Coelho, Beatriz Couto, Sara Dallaspezia, Benjamin A. Ely, Sónia Ferreira, Martine Fontaine, Jean-Paul Fouche, Rachael Grazioplene, Patricia Gruner, Kristen Hagen, Bjarne Hansen, Gregory L. Hanna, Yoshiyuki Hirano, Marcelo Q. Höxter, Morgan Hough, Hao Hu, Chaim Huyser, Toshikazu Ikuta, Neda Jahanshad, Anthony James, Fern Jaspers-Fayer, Selina Kasprzak, Norbert Kathmann, Christian Kaufmann, Minah Kim, Kathrin Koch, Gerd Kvale, Jun Soo Kwon, Luisa Lazaro, Junhee Lee, Christine Lochner, Jin Lu, Daniela Rodriguez Manrique, Ignacio Martínez-Zalacaín, Yoshitada Masuda, Koji Matsumoto, Maria Paula Maziero, Jose M. Menchón, Luciano Minuzzi, Pedro Silva Moreira, Pedro Morgado, Janardhanan C. Narayanaswamy, Jin Narumoto, Ana E. Ortiz, Junko Ota, Jose C. Pariente, Chris Perriello, Maria Picó-Pérez, Christopher Pittenger, Sara Poletti, Eva Real, Y. C. Janardhan Reddy, Daan van Rooij, Yuki Sakai, João Ricardo Sato, Cinto Segalas, Roseli G. Shavitt, Zonglin Shen, Eiji Shimizu, Venkataram Shivakumar, Noam Soreni, Carles Soriano-Mas, Nuno Sousa, Mafalda Machado Sousa, Gianfranco Spalletta, Emily R. Stern, S. Evelyn Stewart, Philip R. Szeszko, Rajat Thomas, Sophia I. Thomopoulos, Daniela Vecchio, Ganesan Venkatasubramanian, Chris Vriend, Susanne Walitza, Zhen Wang, Anri Watanabe, Lidewij Wolters, Jian Xu, Kei Yamada, Je-Yeon Yun, Mojtaba Zarei, Qing Zhao, Xi Zhu, ENIGMA-OCD Working Group, Honami Arai, Ana Isabel Araújo, Kentaro Araki, Paul D. Arnold, Justin T. Baker, Núria Bargalló, Sara Bertolín, John R. Best, Premika S. W. Boedhoe, Sven Bölte, Vilde Brecke, Jan K. Buitelaar, Rosa Calvo, Carolina Cappi, Joao Castelhano, Wei Chen, Sutoh Chihiro, Kang Ik Kevin Cho, Sunah Choi, Daniel Costa, Nan Dai, Shareefa Dalvie, Damiaan Denys, Juliana B. Diniz, Isabel C. Duarte, Calesella Federico, Jamie D. Feusner, Kate D. Fitzgerald, Egill Axfjord Fridgeirsson, Edna Grünblatt, Sayo Hamatani, Gregory Hanna, Mengxin He, Odile A. van den Heuvel, Marcelo Q. Höxter, Morgan Hough, Keisuke Ikari, Jonathan Ipser, Hongyan Jiang, Linling Jiang, Niels T. de Joode, Norbert Kathmann, Taekwan Kim, Hitomi Kitagawa, Masaru Kuno, Yoo Bin Kwak, Jun Soo Kwon, Wieke van Leeuwen, Chiang-shan Ray Li, Na Li, Yanni Liu, Fang liu, Antonio Carlos Lopes, Jin Lu, Yuri Milaneschi, Hein van Marle, Sergi Mas, David Mataix-Cols, Maria Alice de Mathis, Maria Paula Mazieiro, Sarah Medland, Renata Melo, Euripedes C. Miguel, Astrid Morer, Alessandro S. De Nadai, Tomohiro Nakao, Masato Nihei, Luke Norman, Erika L. Nurmi, Joseph O’Neil, Sanghoon Oh, Sho Okawa, John C. Piacentini, Maria Picó-Pérez, Natalia Rodriguez, Daan van Rooij, João R. Sato, Cinto Segalas, Renata Silva, Noam Soreni, Michael Stevens, Anouk van der Straten, Jumpei Takahashi, Tais Tanamatis, Jinsong Tang, Anders Lillevik Thorsen, David Tolin, Anne Uhlmann, Benedetta Vai, Ysbrand D. van der Werf, Dick J. Veltman, Nora Vetter, Jicai Wang, Cees J. Weeland, Guido A. van Wingen, Stella J. de Wit, Nicole Wolff, Xiufeng Xu, Tokiko Yoshida, Fengrui Zhang, Paul M. Thompson, Willem B. Bruin, Guido A. van Wingen, Federica Piras, Fabrizio Piras, Dan J. Stein, Odile A. van den Heuvel, Helen Blair Simpson, Rachel Marsh, Jiook Cha

**Affiliations:** 1 Department of Psychology, College of Social Sciences, Seoul National University, Seoul, Republic of Korea; 2 Department of Brain and Cognitive Sciences, College of Natural Sciences, Seoul National University, Seoul, Republic of Korea; 3 Graduate School of Medical Science, Kyoto Prefectural University of Medicine, Department of Psychiatry, Kyoto City, Japan; 4 Bellvitge Biomedical Research Insitute-IDIBELL, Bellvitge University Hospital, Barcelona, Spain; 5 CIBER of Mental Health (CIBERSAM), Carlos III Health Institute, Madrid, Spain; 6 Department of Clinical Sciences, University of Barcelona, Barcelona, Spain; 7 The Margaret and Wallace McCain Centre for Child, Youth & Family Mental Health and Campbell Family Mental Health Research Institute, Centre for Addiction and Mental Health, Toronto, ON Canada; 8 Department of Psychiatry, University of Toronto, Toronto, ON Canada; 9 Program in Neurosciences and Mental Health, The Hospital for Sick Children, Toronto, ON Canada; 10 Department of Psychiatry, Yale University School of Medicine, New Haven, CT 06510 USA; 11 The Mathison Centre for Mental Health Research & Education, Hotchkiss Brain Institute, Cumming School of Medicine, University of Calgary, Calgary, AB Canada; 12 Departments of Psychiatry and Medical Genetics, Cumming School of Medicine, University of Calgary, Calgary, AB Canada; 13 OCD clinic, Department of Psychiatry, National Institute of Mental Health And Neurosciences (NIMHANS), Bangalore, India; 14 Laboratory of Neuropsychiatry, Department of Clinical and Neuroscience and Neurorehabilitation, IRCCS Santa Lucia Foundation, Rome, Italy; 15 Center of Image Diagnostic, Hospital Clínic de Barcelona, Barcelona, Spain; 16 Magnetic Resonance Image Core Facility, Institut d’Investigacions Biomèdiques August Pi i Sunyer (IDIBAPS), Barcelona, Spain; 17 Departamento e Instituto de Psiquiatria do Hospital das Clinicas, IPQ HCFMUSP, Faculdade de Medicina, Universidade de Sao Paulo, Sao Paulo, SP Brazil; 18 Department of Methods and Techniques in Psychology, Pontifical Catholic University, São Paulo, SP Brazil; 19 Vita-Salute San Raffaele University, Milano, Italy; 20 Psychiatry & Clinical Psychobiology, Division of Neuroscience, IRCCS Scientific Institute Ospedale San Raffaele, Milano, Italy; 21 Bellvitge Biomedical Research Institute-IDIBELL, Bellvitge University Hospital, Barcelona, Spain; 22 Department of Psychology, Humboldt-Universitat zu Berlin, Berlin, Germany; 23 Department of Clinical Neuroscience, Centre for Psychiatric Research and Education, Karolinska Institutet, Stockholm, Sweden; 24 Department of Medical Psychology, Medical School Hamburg, Hamburg, Germany; 25 Department of Child and Adolescent Psychiatry and Psychotherapy, University Hospital of Psychiatry Zurich, University of Zurich, Zurich, Switzerland; 26 Neuroscience Center Zurich, University of Zurich and ETH Zurich, Zurich, Switzerland; 27 McLean Hospital, Belmont, MA USA; 28 Department of Psychiatry, Harvard Medical School, Boston, MA USA; 29 Radboudumc, Department of Cognitive Neuroscience, Nijmegen, The Netherlands; 30 Karakter Child and Adolescent Psychiatry University Center, Nijmegen, The Netherlands; 31 Department of Child and Adolescent Psychiatry and Psychology, Institute of Neurosciences, Hospital Clínic Universitari, Barcelona, Spain; 32 Institut d’Investigacions Biomèdiques August Pi i Sunyer (IDIBAPS), Barcelona, Spain; 33 Department of Medicine, University of Barcelona, Barcelona, Spain; 34 Coimbra Institute for Biomedical Imaging and Translational Research (CIBIT), University of Coimbra, 3000-548 Coimbra, Portugal; 35 Institute for Nuclear Sciences Applied to Health (ICNAS), University of Coimbra, 3000-548 Coimbra, Portugal; 36 Faculty of Medicine, University of Coimbra, 3000-548 Coimbra, Portugal; 37 Department of Psychiatry, First Affiliated Hospital of Kunming Medical University, Kunming, China; 38 Research Center for Child Mental Development, Chiba University, Chiba, Japan; 39 United Graduate School of Child Development, Osaka University, Kanazawa University, Hamamatsu University, Chiba University and University of Fukui, Suita, Japan; 40 Life and Health Sciences Research Institute (ICVS), School of Medicine, University of Minho, Braga, Portugal; 41 ICVS/3B’s, PT Government Associate Laboratory, Braga/Guimaraes, Portugal; 42 Clinical Academic Center - Braga, Braga, Portugal; 43 Psychiatry & Clinical Psychobiology Unit, Division of Neuroscience, Scientific Institute Ospedale San Raffaele, Milano, Italy; 44 Department of Psychiatry and Behavioral Sciences, Albert Einstein College of Medicine, Bronx, NY USA; 45 Columbia University Medical College, Columbia University, New York, NY USA; 46 SAMRC Genomics of Brain Disorders Unit, Department of Psychiatry, Cape Town, South Africa; 47 Hospital of Molde, Møre og Romsdal Hospital Trust, Molde, Norway; 48 Bergen Center for Brain Plasticity, Haukeland University Hospital, Bergen, Norway; 49 Centre for Crisis Psychology, University of Bergen, Bergen, Norway; 50 Department of Psychiatry, University of Michigan Medical School, Ann Arbor, MI USA; 51 Highfield Unit Oxford, Warneford Hospital, Warneford Lane, Headington, Oxford, Oxfordshire OX3 7JX UK; 52 Shanghai Mental Health Center, Shanghai, China; 53 Levvel, academic center for child and adolescent care, Amsterdam, The Netherlands; 54 Department of Child and Adolescent Psychiatry, Amsterdam UMC, Amsterdam, The Netherlands; 55 Department of Communication Sciences and Disorders, University of Mississippi, Oxford, MS USA; 56 Imaging Genetics Center, Mark and Mary Stevens Neuroimaging and Informatics Institute, Keck School of Medicine, University of Southern California, Marina del Rey, Los Angeles, CA USA; 57 Department of Psychiatry University of Oxford, Warneford Hospital, Oxford, OX3 7JX UK; 58 Department of Psychiatry, University of British Columbia, Vancouver, BC Canada; 59 BC Children’s Hospital Research Institute, Vancouver, BC Canada; 60 Amsterdam UMC, Vrije Universteit Amsterdam, Department of Psychiatry, Amsterdam Neuroscience, Amsterdam, The Netherlands; 61 Amsterdam UMC, Vrije Universiteit Amsterdam, Department of Anatomy and Neurosciences, Amsterdam Neuroscience, Amsterdam, The Netherlands; 62 Department of Neuropsychiatry, Seoul National University Hospital, Seoul, Republic of Korea; 63 Department of Psychiatry, Seoul National University College of Medicine, Seoul, Republic of Korea; 64 TUM-Neuroimaging Center (TUM-NIC) of Klinikum rechts der Isar, Technische Universitat Munchen, München, Germany; 65 Department of Diagnostic and Interventional Neuroradiology, School of Medicine, Technical University of Munich, Munich, Germany; 66 Department of Clinical Psychology, University of Bergen, Bergen, Norway; 67 Department of Brain and Cognitive Sciences, Seoul National University College of Natural Sciences, Seoul, Republic of Korea; 68 Institute of Human Behavioral Medicine, SNU-MRC, Seoul, Republic of Korea; 69 Department of Psychiatry, Uijeongbu Eulji Medical Center, Uijeongbu, Republic of Korea; 70 SAMRC Unit on Risk & Resilience in Mental Disorders, Department of Psychiatry, Stellenbosch University, Stellenbosch, South Africa; 71 Department of Psychiatry, First Affiliated Hospitalof Kunming Medical University, Kunming, China; 72 Graduate School of Systemic Neurosciences, Ludwig-Maximilians-University, Munich, Germany; 73 Department of Radiology, Bellvitge University Hospital, Barcelona, Spain; 74 Chiba University Hospital, Chiba University, Chiba, Japan; 75 LIM 23, Instituto de Psiquiatria, Hospital das Clinicas da Faculdade de Medicina da Universidade de Sao Paulo, Sao Paulo, Brazil; 76 Faculty of Medicine, City University of Sao Paulo, Sao Paulo, Brazil; 77 Anxiety Treatment and Research Clinic, St. Joseph’s Hamilton Healthcare, Hamilton, ON Canada; 78 Dapartmente of Psychiatry and Behavioural Neurosciences, McMaster University, Hamilton, ON Canada; 79 Psychological Neuroscience Lab, CIPsi, School of Psychology, University of Minho, Braga, Portugal; 80 Department of Psychiatry, Graduate School of Medical Science, Kyoto Prefectural University of Medicine, Kyoto, Japan; 81 University of Illinois at Urbana-Champaign, Champaign, IL USA; 82 Departamento de Psicología Básica, Clínica y Psicobiología, Universitat Jaume I, Castelló de la Plana, Spain; 83 Department of Psychology, Yale University, New Haven, CT USA; 84 Child Study Center, Yale University, New Haven, CT USA; 85 Center for Brain and Mind Health, Yale University, New Haven, CT USA; 86 Radboud University Medical Center, Donders Institute for Brain, Cognition and Behavior, Department of Cognitive Neuroscience, Nijmegen, The Netherlands; 87 ATR Brain Information Communication Research Laboratory Group, Kyoto, Japan; 88 Center of Mathematics, Computing and Cognition, Universidade Federal do ABC, Santo André, Brazil; 89 Big Data, Hospital Israelita Albert Einstein, São Paulo, Brazil; 90 Departamento de Psiquiatria, Hospital das Clinicas HCFMUSP, Faculdade de Medicina, Universidade de Sao Paulo, Sao Paulo, SP Brazil; 91 Department of Cognitive Behavioral Physiology, Graduate School of Medicine, Chiba University, Chiba, Japan; 92 National Institute of Mental Health and Neurosciences, Department of Integrative Medicine, Bengaluru, India; 93 Department of Psychiatry and Behavioural Neurosciences, McMaster University, Hamilton, ON Canada; 94 Offord Centre for Child Studies, Hamilton, ON Canada; 95 Department of Social Psychology and Quantitative Psychology, University of Barcelona, Barcelona, Spain; 96 Division of Neuropsychiatry, Menninger Department of Psychiatry and Behavioral Science, Baylor College of Medicine, Houston, TX USA; 97 Department of Psychiatry, New York University School of Medicine, New York, NY USA; 98 Clinical Research, Nathan Kline Institute for Psychiatric Research, Orangeburg, NY USA; 99 British Columbia Children’s Hospital, Psychiatry, Vancouver, BC Canada; 100 British Columbia Mental Health and Substance Use Services Research Institute, Vancouver, BC Canada; 101 Departments of Psychiatry and Neuroscience, Icahn School of Medicine at Mount Sinai, New York, NY USA; 102 Mental Illness Research, Education and Clinical Center, James J. Peters VA Medical Center, Bronx, NY USA; 103 Weill-Cornell Medicine Qatar, Education City, Doha, Qatar; 104 Amsterdam Neuroscience, Compulsivity, Impulsivity & Attention program, Amsterdam, The Netherlands; 105 Shanghai Mental Health Center, Shanghai Jiao Tong University School of Medicine, Shanghai, China; 106 Norwegian University of Science and Technology (NTNU), Faculty of Medicine, Regional Centre for Child and Youth Mental Health and Child Welfare (RKBU Central Norway), Klostergata 46, 7030 Trondheim, Norway; 107 Department of Internal Medicine, First Affiliated Hospital of Kunming Medical University, Kunming, China; 108 Department of Radiology, Graduate School of Medical Science, Kyoto Prefectural University of Medicine, Kyoto, Japan; 109 Seoul National University Hospital, Seoul, Republic of Korea; 110 Yeongeon Student Support Center, Seoul National University College of Medicine, Seoul, Republic of Korea; 111 Institute of Medical Science and Technology, Shahid Beheshti University, Tehran, Iran; 112 Department of Psychiatry, Columbia University Irving Medical Center, New York, NY USA; 113 New York State Psychiatric Institute, New York, NY USA; 114 Amsterdam UMC, Universiteit van Amsterdam, Department of Psychiatry, Amsterdam, The Netherlands; 115 Department of Psychiatry and Mental Health, University of Cape Town, Cape Town, South Africa; 116 SAMRC Unit on Risk & Resilience in Mental Disorders, Cape Town, South Africa; 117 CIBERSAM, Barcelona, Spain; 118 Gerontology Research Centre, Simon Fraser University, Burnaby, BC Canada; 119 Amsterdam UMC, Vrije Universiteit Amsterdam, Department of Psychiatry, Department of Anatomy & Neurosciences, Amsterdam, The Netherlands; 120 Department of Women’s & Children’s Health, Center for Psychiatry Research, Karolinska Institutet, Stockholm, Sweden; 121 Magnetic Resonance Image Center, First Affiliated Hospital of Kunming Medical University, Kunming, China; 122 Department of Cognitive Behavioral Physiology, Graduate School of Medicine and School of Medicine, Chiba University, Chiba, Japan; 123 Psychiatry Neuroimaging Laboratory, Department of Psychiatry, Brigham and Women’s Hospital, Harvard Medical School, Boston, MA USA; 124 SA MRC Unit on Risk & Resilience in Mental Disorders, Department of Psychiatry and Mental Health, University of Cape Town, Cape Town, South Africa; 125 Amsterdam UMC, University of Amsterdam, Department of Psychiatry, Amsterdam Neuroscience, Amsterdam, The Netherlands; 126 Division of Neurosciences & Clinical Translation, Temerty Faculty of Medicine, University of Toronto, Toronto, ON Canada; 127 Department of Neuropsychiatry, Graduate School of Medical Sciences, Kyushu University, Fukuoka, Japan; 128 Department of Psychiatry and Mental Health and Neuroscience Institute, Brain Behaviour Unit, University of Cape Town, Cape Town, South Africa; 129 Department of Bio and Brain Engineering, Korea Advanced Institute of Science and Technology, Daejeon, Republic of Korea; 130 Amsterdam UMC, Vrije Universiteit Amsterdam, Department of Psychiatry, Amsterdam Neuroscience, Amsterdam, The Netherlands; 131 Amsterdam UMC, Vrije Universiteit, Department of Psychiatry, Amsterdam Neuroscience, Amsterdam, The Netherlands; 132 Department of Basic Clinical Practice, Pharmacology Unit, University of Barcelona, Barcelona, Spain; 133 IDIBAPS, Barcelona, Spain; 134 Centro de Investigación Biomédica en Red de salud mental (CIBERSAM), Barcelona, Spain; 135 QIMR Berghofer Medical Research Institute, Brisbane, QLD Australia; 136 Department of Child and Adolescent Psychiatry and Psychology, Hospital Clínic of Barcelona. CIBERSAM, Barcelona, Spain; 137 Texas State University, Austin, TX USA; 138 The National Institutes of Health (NIH), Bethesda, MD USA; 139 Department of Psychiatry, University of California, Los Angeles, CA USA; 140 Division of Child and Adolescent Psychiatry, Jane & Terry Semel Institute For Neurosciences, University of California, Los Angeles, CA USA; 141 Pediatric OCD Consultation Clinic, McMaster University, Hamilton, ON Canada; 142 Anxiety Treatment and Research Center, McMaster University, Hamilton, ON Canada; 143 Institute of Living, Hartford, CT USA; 144 Yale University School of Medicine, New Haven, CT USA; 145 Department of Psychiatry, Zhejiang University School of Medicine, Hangzhou, China; 146 Department of Child and Adolescent Psychiatry and Psychotherapy, TU Dresden, Dresden, Germany; 147 Department of Psychology, MSB Medical School Berlin, Berlin, Germany

**Keywords:** Neuroscience, Diagnostic markers

Correction to: *Molecular Psychiatry* 10.1038/s41380-023-02392-6, published online 07 February 2024

In this article Noam Soreni at affiliation ‘Department of Psychiatry and Behavioural Neurosciences, McMaster University, Hamilton, Ontario, Canada and Offord Centre for Child Studies, Hamilton, Ontario, Canada’ was missing from the author list. The original article has been corrected.

