# Supplementary material for: White matter diffusion estimates in obsessive-compulsive disorder across 1653 individuals: machine learning findings from the ENIGMA OCD Working Group
Source: Mol Psychiatry. 2024 Feb 7;29(4):1063–74. doi: 10.1038/s41380-023-02392-6 (PMC11176060; doi:10.1038/s41380-023-02392-6)

Supplementary Materials

## ENIGMA-OCD Working Group Consortium

[**Supplementary Table 1.** Demographic and clinical characteristics of each site.](#_heading=h.32hioqz)

**Supplementary Table 2**. Demographic and clinical characteristics of discovery and replication sets.

[**Supplementary Table 3**. Performance of OCD classification applied NeuroComBat harmonization with covariates (age, sex).](#_heading=h.41mghml)

[**Supplementary Table 4.** Top 10 features of diagnosis classification models](#_heading=h.3tbugp1).

[**Supplementary Table 5.** Top 20 features of medication classification models.](#_heading=h.nmf14n)

[**Supplementary Table 6.** Top 20 features to classify diagnosis derived from LIME.](#_heading=h.1mrcu09)

**Supplementary Figure 1**. Leave-one-site-out cross-validation.

## ENIGMA-OCD Working Group Consortium

Yoshinari Abe ^1^, Pino Alonso ^2, 3, 4^, Stephanie Ameis ^5, 6, 7^, Alan Anticevic ^8^, Honami Arai ^9^, Ana Isabel Araújo^10, 11^, Kentaro Araki ^9^, Paul D. Arnold ^12, 13^, Justin T. Baker ^14, 15^, Srinivas Balachander ^15^, Nerisa Banaj ^16^, Núria Bargalló ^17, 18, 19^, Marcelo C. Batistuzzo ^19, 20^, Francesco Benedetti ^21, 22^, Sara Bertolín ^23. 24^, John R. Best ^25 26^, Jan Carl Beucke ^27, 28, 29^, Premika S.W. Boedhoe ^30^, Irene Bollettini ^22^, Sven Bölte ^31^, Vilde Brecke ^32^, Silvia Brem ^33, 34^, Brian P. Brennan ^13, 14^, Willem B. Bruin ^35, 36^, Jan K. Buitelaar ^38, 39^, Rosa Calvo ^24, 39, 40, 41^, Carolina Cappi ^42^, Joao Castelhano^10, 11^, Miguel Castelo-Branco ^10, 11, 43^, Wei Chen ^44^, Yuqi Cheng ^45^, Ritu Bhusal Chhatkuli ^9, 46^, Sutoh Chihiro ^47^, Kang Ik Kevin Cho ^48, 49^, Sunah Choi ^49^, Valentina Ciullo ^16^, Ana Coelho ^50, 51, 52^, Daniel Costa ^42^, Beatriz Couto ^50, 51, 52^, Nan Dai ^45^, Sara Dallaspezia ^53^, Shareefa Dalvie ^54^, Damiaan Denys ^55^, Juliana B. Diniz ^42^, Isabel C. Duarte ^10, 11^, Benjamin A. Ely ^56^, Calesella Federico ^21^, Sónia Ferreira ^50, 51, 52^, Jamie D. Feusner ^57^, Kate D. Fitzgerald ^58^, Martine Fontaine ^59^, Jean-Paul Fouche ^60^, Egill Axfjord Fridgeirsson ^55^, Rachael Grazioplene ^8^, Edna Grünblatt ^33, 34^, Patricia Gruner ^8^, Kristen Hagen ^32, 61^, Sayo Hamatani ^9, 46^, Gregory Hanna ^58^, Bjarne Hansen ^32, 62^, Mengxin He ^45^, Odile A. van den Heuvel ^36, 63, 64^, Yoshiyuki Hirano ^9, 46^, Marcelo Q. Höxter ^19, 42^, Morgan Hough ^42^, Hao Hu ^66^, Chaim Huyser ^67, 68^, Keisuke Ikari ^69^, Toshikazu Ikuta ^70^, Jonathan Ipser ^71^, Neda Jahanshad ^72^, Anthony James ^73^, Fern Jaspers-Fayer ^74, 75^, Hongyan Jiang ^45^, Linling Jiang ^45^, Niels T. de Joode ^30^, Selina Kasprzak ^63, 64^, Norbert Kathmann ^27^, Christian Kaufmann ^27^, Minah Kim ^76, 77^, Taekwan Kim ^49, 78^, Hitomi Kitagawa ^9^, Kathrin Koch ^79, 80^, Masaru Kuno ^9, 46^, Gerd Kvale ^32, 81^, Yoo Bin Kwak ^49^, Jun Soo Kwon ^49, 77, 82^, Luisa Lazaro ^3, 39, 40, 41^, Junhee Lee ^76, 83^, Wieke van Leeuwen ^55^, Chiang-shan Ray Li ^8^, Na Li ^46^, Yanni Liu ^58^, Fang liu ^45^, Christine Lochner ^84^, Antonio Carlos Lopes ^42^, Jin Lu ^45^, Yuri Milaneschi ^85^, Daniela Rodriguez Manrique ^79, 80, 86^, Hein van Marle ^87^, Rachel Marsh ^59^, Ignacio Martínez-Zalacaín ^2, 88^, Sergi Mas ^89, 90, 91^, Yoshitada Masuda ^92^, David Mataix-Cols ^93^, Maria Alice de Mathis ^94, 95^, Koji Matsumoto ^92^, Maria Paula Mazieiro ^94, 95^, Sarah Medland ^96^, Renata Melo ^42^, Jose M Menchón ^2, 3, 4^, Euripedes C. Miguel ^42^, Luciano Minuzzi ^97, 98^, Pedro Silva Moreira ^50, 51, 99^, Astrid Morer ^90, 100, 101^, Pedro Morgado ^50, 51, 52^, Alessandro S. De Nadai ^102^, Tomohiro Nakao ^69^, Janardhanan C. Narayanaswamy ^15^, Jin Narumoto ^103^, Masato Nihei ^9^, Luke Norman ^104^, Erika L. Nurmi ^105^, Joseph O’Neil ^106^, Sanghoon Oh ^77, 107^, Sho Okawa ^47^, Ana E. Ortiz ^39, 40, 41^, Junko Ota ^9, 46^, Jose C. Pariente ^18^, Chris Perriello ^108^, John C. Piacentini ^106^, Maria Picó-Pérez ^50, 51^, Federica Piras ^16^, Fabrizio Piras ^16^, Christopher Pittenger ^8, 109, 110, 111^, Sara Poletti ^22^, Eva Real ^3, 4^, Y.C.Janardhan Reddy ^15^, Natalia Rodriguez ^89^, Daan van Rooij ^112^, Yuki Sakai ^113, 114^, João R. Sato ^115, 116^, Cinto Segalas ^3, 4^, Roseli G. Shavitt ^42^, Zonglin Shen ^45^, Eiji Shimizu ^9, 46, 117^, Venkataram Shivakumar ^118^, Renata Silva ^19^, H. Blair Simpson ^59^, Noam Soreni ^119, 120^, Carles Soriano-Mas ^3, 4, 121^, Nuno Sousa ^50, 51, 52^, Mafalda Machado Sousa ^50, 51, 52^, Gianfranco Spalletta ^16, 122^, Dan J. Stein ^123, 124^, Emily R. Stern ^125, 126^, Michael Stevens ^127, 128^, S. Evelyn Stewart ^25, 129, 130^, Anouk van der Straten ^55^, Philip R. Szeszko ^131, 132^, Jumpei Takahashi ^9^, Tais Tanamatis ^42^, Jinsong Tang ^133^, Rajat Thomas ^134^, Sophia I. Thomopoulos ^72^, Paul M. Thompson ^72^, Anders Lillevik Thorsen ^32, 62^, David Tolin ^127, 128^, Anne Uhlmann ^135^, Benedetta Vai ^22^, Ysbrand D. van der Werf ^136^, Daniela Vecchio ^16^, Dick J. Veltman ^30^, Ganesan Venkatasubramanian ^15^, Nora Vetter ^137, 138^, Chris Vriend ^36,^ ^63, 64^, Susanne Walitza ^33, 34^, Zhen Wang ^139^, Jicai Wang ^45^, Anri Watanabe ^114^, Cees J. Weeland ^30^, Guido A van Wingen ^35, 36^, Stella J. de Wit ^30^, Nicole Wolff ^137^, Lidewij Wolters ^140^, Jian Xu ^141^, Xiufeng Xu ^45^, Kei Yamada ^142^, Tokiko Yoshida ^9, 46^, Je-Yeon Yun ^143, 144^, Mojtaba Zarei ^145^, Fengrui Zhang ^44^, Qing Zhao ^139^, Xi Zhu ^146, 147^

Affiliation

1. Graduate School of Medical Science, Kyoto Prefectural University of Medicine, Department of Psychiatry, Kyoto City, Japan
2. Bellvitge Biomedical Research Insitute-IDIBELL, Bellvitge University Hospital, Barcelona, Spain
3. CIBER of Mental Health (CIBERSAM), Carlos III Health Institute, Madrid, Spain
4. Department of Clinical Sciences, University of Barcelona, Barcelona, Spain
5. The Margaret and Wallace McCain Centre for Child, Youth & Family Mental Health and Campbell Family Mental Health Research Institute, Centre for Addiction and Mental Health, Toronto, ON, Canada
6. Department of Psychiatry, University of Toronto, Toronto, Ontario
7. Program in Neurosciences and Mental Health, The Hospital for Sick Children, Toronto, ON, Canada
8. Department of Psychiatry, Yale University School of Medicine, New Haven, CT, 06510, USA
9. Research Center for Child Mental Development, Chiba University, Chiba, Japan
10. Coimbra Institute for Biomedical Imaging and Translational Research (CIBIT), University of Coimbra, 3000-548 Coimbra, Portugal
11. Institute for Nuclear Sciences Applied to Health (ICNAS), University of Coimbra, 3000-548 Coimbra, Portugal
12. The Mathison Centre for Mental Health Research & Education, Hotchkiss Brain Institute, Cumming School of Medicine, University of Calgary, Calgary, AB, Canada
13. McLean Hospital, Belmont, MA, USA
14. Department of Psychiatry, Harvard Medical School, Boston, MA, USA
15. OCD clinic, Department of Psychiatry, National Institute of Mental Health And Neurosciences (NIMHANS), Bangalore, India
16. Laboratory of Neuropsychiatry, Department of Clinical and Neuroscience and Neurorehabilitation, IRCCS Santa Lucia Foundation, Rome, Italy
17. Center of Image Diagnostic. Hospital Clínic de Barcelona, Spain
18. Magnetic Resonance Image Core Facility, Institut d'Investigacions Biomèdiques August Pi i Sunyer (IDIBAPS), Spain
19. Departamento e Instituto de Psiquiatria do Hospital das Clinicas, IPQ HCFMUSP, Faculdade de Medicina, Universidade de Sao Paulo, SP, Brazil
20. Department of Methods and Techniques in Psychology, Pontifical Catholic University, São Paulo, SP, Brazil
21. Vita-Salute San Raffaele University, Milano, Italy
22. Psychiatry & Clinical Psychobiology, Division of Neuroscience, IRCCS Scientific Institute Ospedale San Raffaele, Milano
23. Bellvitge Biomedical Research Institute-IDIBELL, Bellvitge University Hospital, Barcelona, Spain
24. CIBERSAM, Barcelona, Spain
25. Department of Psychiatry, University of British Columbia, Vancouver, BC, Canada
26. Gerontology Research Centre, Simon Fraser University, Burnaby, Canada
27. Department of Psychology, Humboldt-Universitat zu Berlin, Berlin, Germany
28. Department of Clinical Neuroscience, Centre for Psychiatric Research and Education, Karolinska Institutet, Stockholm, Sweden
29. Department of Medical Psychology, Medical School Hamburg, Hamburg, Germany
30. Amsterdam UMC, Vrije Universiteit Amsterdam, Department of Psychiatry, Department of Anatomy & Neurosciences, Amsterdam, The Netherlands
31. Department of Women's & Children's Health,Center for Psychiatry Research, Karolinska Institutet, Stockholm, Sweden
32. Bergen Center for Brain Plasticity, Haukeland University Hospital, Bergen, Norway
33. Department of Child and Adolescent Psychiatry and Psychotherapy, University Hospital of Psychiatry Zurich, University of Zurich, Switzerland
34. Neuroscience Center Zurich, University of Zurich and ETH Zurich, Switzerland
35. Amsterdam UMC, Universiteit van Amsterdam, Department of Psychiatry, Amsterdam, The Netherlands
36. Amsterdam Neuroscience, Compulsivity, Impulsivity & Attention program, Amsterdam, The Netherlands
37. Radboudumc, Department of Cognitive Neuroscience, Nijmegen, The Netherlands
38. Karakter Child and Adolescent Psychiatry University Center, Nijmegen, The Netherlands
39. Department of Child and Adolescent Psychiatry and Psychology, Institute of Neurosciences, Hospital Clínic Universitari, Barcelona, Spain
40. Institut d'Investigacions Biomèdiques August Pi i Sunyer (IDIBAPS), Spain
41. Department of Medicine, University of Barcelona, Barcelona, Spain
42. Departamento de Psiquiatria, Hospital das Clinicas HCFMUSP, Faculdade de Medicina, Universidade de Sao Paulo, Sao Paulo, SP, BR.
43. Faculty of Medicine, University of Coimbra, 3000-548 Coimbra, Portugal
44. Magnetic Resonance Image Center, First Affiliated Hospital of Kunming Medical University
45. Department of Psychiatry, First Affiliated Hospital of Kunming Medical University
46. United Graduate School of Child Development, Osaka University, Kanazawa University, Hamamatsu University, Chiba University and University of Fukui, Suita, Japan
47. Department of Cognitive Behavioral Physiology, Graduate School of Medicine and School of Medicine, Chiba University, Chiba, Japan
48. Psychiatry Neuroimaging Laboratory, Department of Psychiatry, Brigham and Women's Hospital, Harvard Medical School, Boston, MA, USA
49. Department of Brain and Cognitive Sciences, College of Natural Sciences, Seoul National University, Seoul, Republic of Korea
50. Life and Health Sciences Research Institute (ICVS), School of Medicine, University of Minho, Braga, Portugal
51. ICVS/3B's, PT Government Associate Laboratory, Braga/Guimaraes, Portugal.
52. Clinical Academic Center - Braga, Braga, Portugal
53. Psychiatry & Clinical Psychobiology Unit, Division of Neuroscience, Scientific Institute Ospedale San Raffaele, Milano, Italy
54. SA MRC Unit on Risk & Resilience in Mental Disorders, Department of Psychiatry and Mental Health, University of Cape Town, Cape Town, South Africa
55. Amsterdam UMC, University of Amsterdam, Department of Psychiatry, Amsterdam Neuroscience, Amsterdam, The Netherlands
56. Department of Psychiatry and Behavioral Sciences, Albert Einstein College of Medicine, Bronx, NY, United States
57. Division of Neurosciences & Clinical Translation, Temerty Faculty of Medicine, University of Toronto, Toronto, ON, Canada
58. Department of Psychiatry, University of Michigan Medical School, Ann Arbor, MI
59. Columbia University Medical College, Columbia University, New York, NY, U.S.A.
60. SAMRC Genomics of Brain Disorders Unit, Department of Psychiatry, Cape Town, South Africa
61. Hospital of Molde, Møre og Romsdal Hospital Trust, Molde, Norway
62. Centre for Crisis Psychology, University of Bergen, Bergen, Norway
63. Amsterdam UMC, Vrije Universteit Amsterdam, Department of Psychiatry, Amsterdam Neuroscience, Amsterdam, The Netherlands
64. Amsterdam UMC, Vrije Universiteit Amsterdam, Department of Anatomy and Neurosciences, Amsterdam Neuroscience, Amsterdam, The Netherlands
65. Highfield Unit Oxford, Warneford Hospital, Warneford Lane, Headington, Oxford, Oxfordshire, OX3 7JX
66. Shanghai Mental Health Center
67. Levvel, academic center for child and adolescent care, Amsterdam, The Netherlands
68. Department of Child and Adolescent Psychiatry, Amsterdam UMC, Amsterdam, The Netherlands
69. Department of Neuropsychiatry, Graduate School of Medical Sciences, Kyushu University
70. Department of Communication Sciences and Disorders, University of Mississippi, MS, USA
71. Department of Psychiatry and Mental Health and Neuroscience Institute, Brain Behaviour Unit, University of Cape Town, Cape Town, South Africa
72. Imaging Genetics Center, Mark and Mary Stevens Neuroimaging and Informatics Institute, Keck School of Medicine, University of Southern California, Marina del Rey, California, USA
73. Department of Psychiatry University of Oxford, Warneford Hospital, OX3 7JX, UK
74. Department of Psychiatry, University of British Columbia, BC, Canada
75. BC Children's Hospital Research Institute, BC, Canada
76. Department of Neuropsychiatry, Seoul National University Hospital, Seoul, Republic of Korea
77. Department of Psychiatry, Seoul National University College of Medicine, Seoul, Republic of Korea
78. Department of Bio and Brain Engineering, Korea Advanced Institute of Science and Technology, Daejeon, Republic of Korea
79. TUM-Neuroimaging Center (TUM-NIC) of Klinikum rechts der Isar, Technische Universitat Munchen, Germany
80. Department of Diagnostic and Interventional Neuroradiology, School of Medicine, Technical University of Munich
81. Department of Clinical Psychology, University of Bergen, Norway
82. Institute of Human Behavioral Medicine, SNU-MRC, Seoul, Republic of Korea
83. Department of Psychiatry, Uijeongbu Eulji Medical Center, Uijeongbu, Republic of Korea
84. SAMRC Unit on Risk & Resilience in Mental Disorders, Department of Psychiatry, Stellenbosch University, Stellenbosch, South Africa
85. Amsterdam UMC, Vrije Universiteit Amsterdam, Department of Psychiatry, Amsterdam Neuroscience, Amsterdam, The Netherlands
86. Graduate School of Systemic Neurosciences, Ludwig-Maximilians-University, Munich
87. Amsterdam UMC, Vrije Universiteit, Department of Psychiatry, Amsterdam Neuroscience, Amsterdam, The Netherlands
88. Department of Radiology, Bellvitge University Hospital, Barcelona, Spain
89. Department of Basic Clinical Practice, Pharmacology Unit, University of Barcelona, Spain
90. IDIBAPS
91. Centro de Investigación Biomédica en Red de salud mental (CIBERSAM), Spain
92. Chiba University Hospital, Chiba University, Chiba, Japan
93. Sweden Department of Clinical Neuroscience, Centre for Psychiatric Research and Education, Karolinska Institutet, Stockholm, Sweden
94. LIM 23, Instituto de Psiquiatria, Hospital das Clinicas da Faculdade de Medicina da Universidade de Sao Paulo, Sao Paulo, Brazil
95. Faculty of Medicine, City University of Sao Paulo, Sao Paulo, Brazil
96. QIMR Berghofer Medical Research Institute, Brisbane, QLD, Australia
97. Anxiety Treatment and Research Clinic, St. Joseph's Hamilton Healthcare, Hamilton, Ontario, Canada
98. Dapartmente of Psychiatry and Behavioural Neurosciences, McMaster University, Hamilton, Ontario, Canada
99. Psychological Neuroscience Lab, CIPsi, School of Psychology, University of Minho, Braga, Portugal
100. Department of Child and Adolescent Psychiatry and Psychology, Hospital Clínic of Barcelona, Spain. CIBERSAM
101. Department of Medicine. University of Barcelona, Spain
102. Texas State University, Austin, TX, USA
103. Department of Psychiatry, Graduate School of Medical Science, Kyoto Prefectural University of Medicine
104. The National Institutes of Health (NIH)
105. Department of Psychiatry, University of California, Los Angeles, CA, USA
106. Division of Child and Adolescent Psychiatry, Jane & Terry Semel Institute For Neurosciences, University of California, Los Angeles, CA, USA
107. Department of Psychiatry, Uijeongbu Eulji Medical Center, Uijeongbu, Republic of Korea.
108. University of Illinois at Urbana-Champaign, Champaign, IL, USA
109. Department of Psychology, Yale University, New Haven, CT
110. Child Study Center, Yale University, New Haven, CT
111. Center for Brain and Mind Health, Yale University, New Haven, CT
112. Radboud University Medical Center, Donders Institute for Brain, Cognition and Behavior, Department of Cognitive Neuroscience, Nijmegen, The Netherlands.
113. ATR Brain Information Communication Research Laboratory Group, Kyoto, Japan
114. Department of Psychiatry, Graduate School of Medical Science, Kyoto Prefectural University of Medicine, Kyoto, Japan
115. Center of Mathematics, Computing and Cognition, Universidade Federal do ABC, Santo André, Brazil.
116. Big Data, Hospital Israelita Albert Einstein, São Paulo, Brazil
117. Department of Cognitive Behavioral Physiology, Graduate School of Medicine, Chiba University, Chiba, Japan
118. National Institute of Mental Health and Neurosciences, Department of Integrative Medicine, Bengaluru, India
119. Pediatric OCD Consultation Clinic, McMaster University, Hamilton, Ontario, Canada
120. Anxiety Treatment and Research Center, McMaster University, Hamilton, Ontario, Canada
121. Department of Social Psychology and Quantitative Psychology, University of Barcelona, Barcelona, Spain
122. Division of Neuropsychiatry, Menninger Department of Psychiatry and Behavioral Science, Baylor College of Medicine, Houston, TX, USA
123. Department of Psychiatry and Mental Health, University of Cape Town, South Africa
124. SAMRC Unit on Risk & Resilience in Mental Disorders, South Africa
125. Department of Psychiatry, New York University School of Medicine, New York, NY
126. Clinical Research, Nathan Kline Institute for Psychiatric Research, Orangeburg, NY
127. Institute of Living, Hartford, CT, USA
128. Yale University School of Medicine, New Haven, USA
129. British Columbia Children's Hospital Research Institue, Vancouver, BC, Canada
130. British Columbia Mental Health and Substance Use Services Research Institute, Vancouver, BC, Canada
131. Departments of Psychiatry and Neuroscience, Icahn School of Medicine at Mount Sinai, NY, NY USA
132. Mental Illness Research, Education and Clinical Center, James J. Peters VA Medical Center, Bronx, NY USA
133. Department of Psychiatry, Zhejiang University School of Medicine, Hangzhou, China
134. Weill-Cornell Medicine Qatar, Education City, Doha
135. Department of Child and Adolescent Psychiatry and Psychotherapy, TU Dresden, Dresden, Germany
136. Amsterdam UMC, Vrije Universiteit Amsterdam, Department of Anatomy & Neurosciences, Amsterdam Neuroscience, Amsterdam, The Netherlands
137. Department of Child and Adolescent Psychiatry and Psychotherapy, TU Dresden, Dresden, Germany
138. Department of Psychology, MSB Medical School Berlin, Berlin, Germany
139. Shanghai Mental Health Center, Shanghai Jiao Tong University School of Medicine
140. Norwegian University of Science and Technology (NTNU), Faculty of Medicine, Regional Centre for Child and Youth Mental Health and Child Welfare (RKBU Central Norway), Klostergata 46, 7030 Trondheim, Norway
141. Department of Internal Medicine, First Affiliated Hospital of Kunming Medical University
142. Department of Radiology, Graduate School of Medical Science, Kyoto Prefectural University of Medicine
143. Seoul National University Hospital, Seoul, Republic of Korea
144. Yeongeon Student Support Center, Seoul National University College of Medicine, Seoul, Republic of Korea
145. Institute of Medical Science and Technology, Shahid Beheshti University, Tehran, Iran
146. Department of Psychiatry, Columbia University Irving Medical Center, New York, USA
147. New York State Psychiatric Institute, New York, USA

## Supplementary Table 1. Demographic and clinical characteristics of each site.

| **Site** | **OCD/HC (N)** | **Age** | **Male** | **Medicated N (%)** | **Childhood-onset N (%)** | **Duration of illness** | **YBOCS score** | **Lifetime anxiety N (%)** | **Lifetime depression N (%)** |
| --- | --- | --- | --- | --- | --- | --- | --- | --- | --- |
| **(a) Adult** | | | | | | | | | |
| **Amsterdam** | 38/34 | 39.2±10.5 | 16 (42.1) | 0 | 24 (66.7) | 23.7 ± 12.8 | 21.3 ± 6.1 | 16 (42.1) | 18 (47.4) |
| **Bangalore** | 158/131 | 28.1±6.2 | 90 (57) | 63 (40) | 47 (29.7) | 7.2 ± 5.2 | 25.5 ± 6.5 | 14 (8.7) | 21 (13.3) |
| **Capetown** | 22/26 | 30±10.2 | 11 (47.8) | 9 (39.1) | 17 (73.9) | 17.2 ± 11.5 | 23 ± 4.2 | 0 (0) | 0 |
| **Kyoto** | 35/41 | 31.3±8.7 | 14 (40) | 0 | 10 (28.6) | 7.7 ± 6.2 | 21.9 ± 6.6 | 3 (8.6) | 0 |
| **Milan** | 63/65 | 34.3±11.4 | 44 (69.8) | 38 (60.3) | 41 (65.1) | 18.9 ± 11.6 | 31.4 ± 5.2 | 1 (1.6) | 5 (7.9) |
| **NYC** | 16/18 | 27.9±6.9 | 5 (31.3) | 13 (81.3) | 13 (81.3) | 15.1 ± 6.7 | 19.9 ± 5.9 | 8 (50) | 3 (18.8) |
| **Munich** | 73/60 | 31.1±9.6 | 47 (64.4) | 44 (60.3) | 41 (56.9) | 13.5 ± 10.3 | 20.8 ± 6.2 | 0 (0) | 0 |
| **Rome** | 77/111 | 36.5±11 | 50 (65.8) | 68 (89.5) | 42 (56.8) | 17.3 ± 12.8 | 23.2 ± 9.3 | 8 (10.5) | 7 (9.2) |
| **Sao Paulo** | 37/29 | 36.3±11.7 | 16 (43.2) | 13 (35.1) | 31 (88.6) | 26.3 ± 13.5 | 29.2 ± 6.2 | 25 (67.6) | 28 (75.7) |
| **Seoul** | 92/86 | 26±6.5 | 60 (65.2) | 13 (14.1) | 41 (46.1) | 6.2 ± 7 | 25.8 ± 6.9 | 1 (1.1) | 2 (2.17) |
| **Shanghai** | 79/45 | 29.2±9.2 | 44 (55.7) | 0 | 21 (26.9) | 6 ± 5.9 | 26.2 ± 4.7 | 0 (0) | 0 |
|  | | | | | | | | | |
| **Bangalore** | 13/12 | 13.7±2.0 | 6 (23.0) | 11 (42.3) |  | 1.46±1.0 | 21±7.6 | 3 (23.1) | 1 (7.7) |
| **Barcelona** | 52/27 | 14.9±1.8 | 30 (28.9) | 41 (39.4) |  | 2.64±2.2 | 18.7±7.6 | 15 (28.9) | 3 (5.8) |
| **British Columbia** | 13/16 | 13.3±3.2 | 3 (11.5) | 11 (42.3) |  | 3.12±2.7 | 13.4±6.4 | 15 (38.5) | 0 |
| **Calgary** | 19/18 | 12.2±2.4 | 10 (26.3) | 0 |  |  | 23.1±4.7 | NA | 0 |
| **Chiba** | 20/6 | 14.3±2.0 | 13 (32.5) | 8 (20) |  | 2.1±1.8 | 26.9±6.2 | 2 (10) | 0 |
| **Oxford** | 21/23 | 16.3±1.3 | 11 (26.2) | 14 (33.3) |  | 4.46±3.2 | 19.6±7.4 | 7 (33.3) | 5 (23.8) |
| **Yale** | 23/22 | 14.3±2.2 | 13 (26.3) | 12 (28.6) |  |  | 26.9±4.5 | 10 (43.5) | 9 (39.1) |
| **Zurich** | 14/18 | 15.2±1.5 | 11 (27.7) | 8 (30.0) |  | 4.74±2.3 | 16.1±10.2 | 6 (42.9) | 0 |
| *YBOCS* Yale-Brown Obsessive–Compulsive Scale, *NA* not available. | | | | | | | | | |

##

## Supplementary Table 2. Demographic and clinical characteristics of discovery and replication sets. (A), Adult sample to classify OCD vs. HC. (B), Adult sample to classify unmedicated OCD vs. HC. (C), Adult sample to classify medicated OCD vs. unmedicated OCD. (D) Pediatric sample to classify OCD vs. HC. (E), Pediatric sample to classify unmedicated OCD vs. HC. (F), Pediatric sample to classify medicated OCD vs. unmedicated OCD.

## Adult sample to classify OCD vs. HC.

| **Characteristics** | **Train sample for diagnosis in adults** | | **Test sample for diagnosis in adults** | |
| --- | --- | --- | --- | --- |
|  | **OCD sample** | **HC sample** | **OCD sample** | **HC sample** |
|  | **(n = 552)** | **(n = 516)** | **(n = 138)** | **(n = 130)** |
| Age (years) | 31.6± 9.78 | 30.8± 9.97 | 30.9±9.71 | 32.1±10.5 |
| OCD illness severity score | 24.9± 7.14 |  | 25.5±7.02 |  |
| Duration of illness | 12.5± 11.1 |  | 11.8±10.7 |  |
|  | N (%) | N (%) | N (%) | N (%) |
| Male | 314 (58.5) | 302 (56.9) | 83 (60.14) | 78 (60) |
| Medication use at time of scan | 207 (61.8) |  | 54 (39.13) |  |
| Childhood-onset | 258 (47.3) |  | 70 (52.2) |  |
| Lifetime diagnosis |  |  |  |  |
| Anxiety | 61 (73.6) |  | 15(10.87) |  |
| Major depression | 70 (12.7) |  | 14 (10.14) |  |
| Current comorbid disorders |  |  |  |  |
| Anxiety | 55 (10.0) |  | 14 (10.14) |  |
| Major depression | 66 (12.0) |  | 11 (7.97) |  |
| OCD symptom dimension |  |  |  |  |
| Aggressive/checking | 328 (59.42) |  | 83 (60.14) |  |
| Contamination/cleaning | 281 (50.91) |  | 74 (53.62) |  |
| Symmetry/ordering | 299 (54.17) |  | 71 (51.45) |  |
| Sexual/religious | 181 (32.79) |  | 47 (34.06) |  |
| Hoarding | 92 (16.7) |  | 22 (15.94) |  |

## Adult sample to classify unmedicated OCD vs. HC.

| **Characteristics** | **Train sample for classification of unmedicated OCD from HC in adults** | | **Test sample for unmedicated OCD from HC in adults** | |
| --- | --- | --- | --- | --- |
|  | **unmedicated OCD sample** | **HC sample** | **unmedicated OCD sample** | **HC sample** |
|  | **(n = 338)** | **(n = 516)** | **(n = 85)** | **(n = 129)** |
| Age (years) | 31.4± 9.61 | 30.5± 9.8 | 29.5±8.26 | 32.1±10.5 |
| OCD illness severity score | 25± 6.39 |  | 25.2±6.5 |  |
| Duration of illness | 11.4± 11.0 |  | 8.6±8.9 |  |
|  | N (%) | N (%) | N (%) | N (%) |
| Male | 183 (54.14) | 303 (58.6) | 48 (56.5) | 77 (59.7) |
| Medication use at time of scan | 338 (100) |  | 85 (100) |  |
| Childhood-onset | 149 (44.5) |  | 32 (37.6) |  |
| Lifetime diagnosis |  |  |  |  |
| Anxiety | 36 (10.65) |  | 11 (12.94) |  |
| Major depression | 38 (11.24) |  | 9 (10.59) |  |
| Current comorbid disorders |  |  |  |  |
| Anxiety | 33 (9.8) |  | 10 (11.76) |  |
| Major depression | 21 (6.2) |  | 7 (8.24) |  |
| OCD symptom dimension |  |  |  |  |
| Aggressive/checking | 203 (60.06) |  | 43 (50.59) |  |
| Contamination/cleaning | 162 (47.93) |  | 39 (45.88) |  |
| Symmetry/ordering | 201 (59.47) |  | 50 (58.82) |  |
| Sexual/religious | 112 (33.14) |  | 13 (15.29) |  |
| Hoarding | 54 (15.98) |  | 20 (23.53) |  |

## Adult sample to classify medicated OCD vs. unmedicated OCD.

| **Characteristics** | **Train sample for classification of medicated OCD from unmedicated OCD in adults** | | **Test sample for medicated OCD from unmedicated OCD in adults** | |
| --- | --- | --- | --- | --- |
|  | **medicated OCD sample** | **unmedicated OCD sample** | **medicated OCD sample** | **unmedicated OCD**  **sample** |
|  | **(n = 209)** | **(n = 228)** | **(n = 52)** | **(n = 85)** |
| Age (years) | 32.4± 10 | 31± 9.31 | 32.9±10.3 | 30.7±9.68 |
| OCD illness severity score | 24.8± 8.2 | 25.1± 6.38 | 25.1±8.02 | 24.8±6.5 |
| Duration of illness | 15.2± 11.3 | 10.8± 10.4 | 13.8±10.8 | 10.8±11.7 |
|  | N (%) | N (%) | N (%) | N (%) |
| Male | 129 (61.72) | 184 (84.44) | 33 (63.46) | 47 (55.3) |
| Medication use at time of scan | 209 (100) |  | 85 (100) | 85 (100) |
| Childhood-onset | 266 (49.4) |  | 60 (44.1) |  |
| Lifetime diagnosis |  |  |  |  |
| Anxiety | 22 (10.53) | 35 (10.36) | 5 (9.62) | 12 (14.12) |
| Major depression | 25 (11.96) | 32 (9.47) | 11 (21.15) | 15 (17.65) |
| Current comorbid disorders |  |  |  |  |
| Anxiety | 20 (9.57) | 31 (9.17) | 5 (9.62) | 12 (14.12) |
| Major depression | 36 (17.22) | 19 (5.62) | 12 (23.08) | 9 (10.59) |
| OCD symptom dimension |  |  |  |  |
| Aggressive/checking | 128 (61.24) | 200 (59.17) | 33 (63.46) | 46 (54.12) |
| Contamination/cleaning | 119 (56.94) | 165 (48.82) | 32 (61.54) | 36 (42.35) |
| Symmetry/ordering | 86 (41.15) | 195 (57.69) | 30 (57.69) | 56 (65.88) |
| Sexual/religious | 82 (39.23) | 99 (29.29) | 18 (34.62) | 26 (30.59) |
| Hoarding | 29 (13.88) | 58 (17.16) | 9 (17.31) | 16 (18.82) |

## Pediatric sample to classify OCD vs. HC.

| **Characteristics** | **Train sample for diagnosis in pediatrics** | | **Test sample for diagnosis in pediatrics** | |
| --- | --- | --- | --- | --- |
|  |  |  |  |  |
|  | **OCD sample** | **HC sample** | **OCD sample** | **HC sample** |
|  | **(n = 113)** | **(n = 140)** | **(n = 35)** | **(n = 29)** |
| Age (years) | 14.5± 2.24 | 14.4± 2.48 | 14.6±2.35 | 14.2±2.34 |
| OCD illness severity score | 20.8± 8.03 |  | 21.0±8.12 |  |
| Duration of illness | 3.00± 2.53 |  | 2.98±2.29 |  |
|  | N (%) | N (%) | N (%) | N (%) |
| Male | 77 (55) | 20 (59.14) | 83 (60.14) | 78 (60) |
| Medication use at time of scan | 81 (57.86) |  | 24 (68.57) |  |
| Lifetime diagnosis |  |  |  |  |
| Anxiety | 38 (27.14) |  | 10 (28.57) |  |
| Major depression | 17 (76.43) |  | 1 (2.86) |  |
| Current comorbid disorders |  |  |  |  |
| Anxiety | 21 (15.0) |  | 8 (22.86) |  |
| Major depression | 21 (15.0) |  | 8 (22.86) |  |
| OCD symptom dimension |  |  |  |  |
| Aggressive/checking | 14 (10.0) |  | 1 (2.86) |  |
| Contamination/cleaning | 14 (10.0) |  | 2 (5.71) |  |
| Symmetry/ordering | 7 (5.0) |  | 0 (0) |  |
| Sexual/religious | 0 (0) |  | 0 (0) |  |
| Hoarding | 5 (3.57) |  | 1 (2.86) |  |

## Pediatric sample to classify unmedicated OCD vs. HC.

| **Characteristics** | **Train sample for classification of unmedicated OCD from HC in pediatrics** | | **Test sample for unmedicated OCD from HC in pediatrics** | |
| --- | --- | --- | --- | --- |
|  | **unmedicated OCD sample** | **HC sample** | **unmedicated OCD sample** | **HC sample** |
|  | **(n = 56)** | **(n = 95)** | **(n = 14)** | **(n = 24)** |
| Age (years) | 13.3± 2.31 | 13,9± 2.36 | 14.5±2.32 | 14.1±2.77 |
| OCD illness severity score | 20.8± 8.10 |  | 17.7±8.91 |  |
| Duration of illness | 2.82± 2.47 |  | 2.9±2.44 |  |
|  | N (%) | N (%) | N (%) | N (%) |
| Male | 33 (58.93) | 52 (54.74) | 6 (42.86) | 13 (54.17) |
| Medication use at time of scan | 0 (0.0) |  | 0 (0.0) |  |
| Lifetime diagnosis |  |  |  |  |
| Anxiety | 10 (17.86) |  | 2 (14.29) |  |
| Major depression | 2 (3.57) |  | 2 (14.29) |  |
| Current comorbid disorders |  |  |  |  |
| Anxiety | 3 (5.36) |  | 1 (7.14) |  |
| Major depression | 0 (0.0) |  | 0 (0.0) |  |
| OCD symptom dimension |  |  |  |  |
| Aggressive/checking | 7 (12.5) |  | 2 (21.43) |  |
| Contamination/cleaning | 8 (14.29) |  | 1 (7.14) |  |
| Symmetry/ordering | 3 (5.36) |  | 0 (0.0) |  |
| Sexual/religious | 0 (0.0) |  | 0 (0.0) |  |
| Hoarding | 0 (0.0) |  | 0 (0.0) |  |

1. **Pediatric sample to classify medicated OCD vs. unmedicated OCD.**

| **Characteristics** | **Train sample for classification of medicated OCD from unmedicated OCD in pediatrics** | | **Test sample for medicated OCD from unmedicated OCD in pediatrics** | |
| --- | --- | --- | --- | --- |
|  | **medicated OCD sample** | **unmedicated OCD sample** | **medicated OCD sample** | **unmedicated OCD sample** |
|  | **(n = 84)** | **(n = 56)** | **(n = 21)** | **(n = 14)** |
| Age (years) | 15.2± 1.9 | 13.4± 2.4 | 14.5±2.24 | 14.1±2.13 |
| OCD illness severity score | 21.8± 8.10 | 19.7± 8.66 | 18.9±6.42 | 22.2±6.56 |
| Duration of illness | 2.96± 8.10 | 2.65± 1.90 | 18.9±6.42 | 3.89±4.54 |
|  | N (%) | N (%) | N (%) | N (%) |
| Male | 47 (55.95) | 29 (51.79) | 11 (52.38) | 10 (71.43) |
| Medication use at time of scan | 84 (100) |  | 21 (100) |  |
| Lifetime diagnosis |  |  |  |  |
| Anxiety | 56 (55.36) | 31 (55.36) | 8 (38.1) | 1 (7.14) |
| Major depression | 11 (13.10) | 4 (7.14) | 3 (14.29) | 0 (0.0) |
| Current comorbid disorders |  |  |  |  |
| Anxiety | 19 (22.62) | 4 (7.14) | 6 (28.57) | 0 (0.0) |
| Major depression | 5 (70.24) | 0 (0.0) | 1 (4.76) | 0 (0.0) |
| OCD symptom dimension |  |  |  |  |
| Aggressive/checking | 5 (5.95) | 6 (10.71) | 1 (4.76) | 3 (21.43) |
| Contamination/cleaning | 6 (45.24) | 7 (12.5) | 1 (4.76) | 2 (14.29) |
| Symmetry/ordering | 4 (40.48) | 2 (35.71) | 0 (0.0) | 1 (7.14) |
| Sexual/religious | 0 (0.0) | 0 (0.0) | 0 (0.0) | 0 (0.0) |
| Hoarding | 1 (1.19) | 3 (5.36) | 1 (4.76) | 1 (7.14) |

## Supplementary Table 3. Performance of OCD classification applied NeuroComBat harmonization with covariates (age, sex). (95% confidence interval) (A) Adult sample applied NeuroComBat with covariates (age, sex), (B) Pediatric sample applied NeuroComBat with covariates (age, sex).

## Adult sample applied NeuroComBat with covariates (age, sex)

|  | **OCD (N = 690)  vs. HC (N = 646)** | | **unmedicated OCD (N = 429) vs. HC (N = 646)** | | **unmedicated OCD (N = 429)  vs. medicated OCD (N = 261)** | |
| --- | --- | --- | --- | --- | --- | --- |
|  | **Discovery set** | **Replication set** | **Discovery set** | **Replication set** | **Discovery set** | **Replication set** |
| ROC-AUC | 61.61 ± 6.53 | 68.27 ± 3.74 | 67.2 ± 5.82 | 51.35 ± 4.11 | 64.36 ± 9.31 | 61.76 ± 5.02 |
| Accuracy (%) | 63.1 ± 4.69 | 69.63 ± 3.10 | 68.9 ± 6.28 | 55.6 ± 3.83 | 63.26 ± 10.02 | 63.5 ± 5.20 |
| Sensitivity (%) | 64.4 ± 14.44 | 51.8 ± 19.80 | 76.21 ± 8.46 | 56.47 ± 11.39 | 55.44 ± 19.11 | 50 ± 21.58 |
| Specificity (%) | 61.67 ± 17.46 | 81.4 ± 3.98 | 62.82 ± 16.88 | 55.04 ± 14.62 | 82.44 ± 7.68 | 71.8 ± 11.99 |
| For the classification of medication status among OCD patients, some sites (i.e., Amsterdam, Shanghai) containing only unmedicated OCD were excluded from the discovery set. | | | | | | |

**(B) Pediatric sample, NeuroComBat with covariates (age, sex)**

|  | **OCD (N = 175)  vs. HC (N = 142)** | | **unmedicated OCD (N = 70)  vs. HC (N = 142)** | | **unmedicated OCD (N = 105)  vs. medicated OCD (N = 70)** | |
| --- | --- | --- | --- | --- | --- | --- |
|  | **Discovery set** | **Replication set** | **Discovery set** | **Replication set** | **Discovery set** | **Replication set** |
| ROC-AUC | 53.85 ± 11.71 | 63.74 ± 7.12 | 58.02 ± 13.41 | 44.05 ± 10.79 | 63.24 ± 0.34 | 54.76 ± 10.28 |
| Accuracy (%) | 60.15 ± 9.87 | 64.06 ± 6.40 | 58.32 ± 12.80 | 68.42 ± 4.02 | 65.65 ± 0.36 | 60 ± 8.72 |
| Sensitivity (%) | 51.7 ± 22.96 | 65.7 ± 14.60 | 76.83 ± 11.82 | 14.29 ± 43.73 | 70.2 ± 1.69 | 52.38 ± 19.45 |
| Specificity (%) | 69.06 ± 14.87 | 62.07 ± 17.62 | 59.22 ± 2.26 | 100 | 68.96 ± 2.15 | 71.43 ± 14.58 |
| For the classification of medication status among OCD patients, some sites (i.e., Calgary) containing only unmedicated OCD were excluded from the discovery set. | | | | | | |

##

**Supplementary Table 4. Top 10 features of diagnosis classification models.** (A) Classification of OCD from HC in adult samples (OCD: N = 690, HC: N = 646). (B) Classification of OCD from HC in pediatric samples (OCD: N = 175, HC: N = 142). (C) Classification of unmedicated OCD from HC in adult samples (unmedicated OCD: N = 429, HC: N = 646). (D) Classification of unmedicated OCD from HC in pediatric samples (unmedicated OCD: N = 70, HC: N = 142)

## To further investigate the effect of diagnosis and medication effect, we applied a machine learning interpretation method, K-LIME (K-Local Interpretable Model-agnostic Explanation), to the classification models. These interpretation models accounted for the variance of the best machine learning models ranging from 45.69% to 63.8% (Supplementary Table 4, 5)

1. **Classification of OCD from HC in adult samples** (OCD: N = 690, HC: N = 646)

| **Rank** | **Feature** | **DTI Metric** | **Weight** |
| --- | --- | --- | --- |
| 1 | Right Superior corona radiata | MD | 1.000 |
| 2 | Age | . | 0.997 |
| 3 | Left Posterior thalamic radiation | FA | 0.978 |
| 4 | Genu of corpus callosum | MD | 0.900 |
| 5 | Bilateral Posterior thalamic radiation | FA | 0.863 |
| 6 | Left Sagittal stratum | FA | 0.716 |
| 7 | Bilateral Posterior limb of internal capsule | FA | 0.703 |
| 8 | Bilateral Superior longitudinal fasciculus | FA | 0.670 |
| 9 | Left Posterior limb of internal capsule | AD | 0.660 |
| 10 | Bilateral Superior longitudinal fasciculus | MD | 0.617 |

**(B) Classification of OCD from HC in pediatric samples** (OCD: N = 175, HC: N = 142)

| **Rank** | **Feature** | **DTI Metric** | **Weight** |
| --- | --- | --- | --- |
| 1 | Left Cingulum (hippocampus) | MD | 1.000 |
| 2 | Bilateral Uncinate fasciculus | MD | 0.886 |
| 3 | Right Fornix and Stria terminalis | FA | 0.833 |
| 4 | Right External capsule | AD | 0.829 |
| 5 | Bilateral Cingulum (hippocampus) | AD | 0.815 |
| 6 | Right Corticospinal tract | FA | 0.788 |
| 7 | Left Anterior corona radiata | AD | 0.778 |
| 8 | Left Corticospinal tract | FA | 0.730 |
| 9 | Bilateral Fornix and Stria terminalis | FA | 0.715 |
| 10 | Right Retrolenticular part of internal capsule | MD | 0.707 |

**(C) Classification of unmedicated OCD from HC in adult sample** (unmedicated OCD: N = 429, HC: N = 646)

| **Rank** | **Feature** | **DTI Metric** | **Weight** |
| --- | --- | --- | --- |
| 1 | Age | . | 1.000 |
| 2 | Bilateral Fornix and Stria terminalis | RD | 0.908 |
| 3 | Genu of corpus callosum | RD | 0.880 |
| 4 | Right Posterior limb of internal capsule | FA | 0.748 |
| 5 | Right Inferior fronto-occipital fasciculus | AD | 0.739 |
| 6 | Bilateral Posterior limb of internal capsule | FA | 0.696 |
| 7 | Corpus Callosum | AD | 0.660 |
| 8 | Left Posterior limb of internal capsule | AD | 0.642 |
| 9 | Left Posterior limb of internal capsule | FA | 0.638 |
| 10 | Right Uncinate fasciculus | AD | 0.459 |

**(D) Classification of unmedicated OCD from HC in pediatric samples** (unmedicated OCD: N = 70, HC: N = 142)

| **Rank** | **Feature** | **DTI Metric** | **Weight** |
| --- | --- | --- | --- |
| 1 | Splenium of corpus callosum | FA | 1.000 |
| 2 | Bilateral Retrolenticular part of internal capsule | FA | 0.826 |
| 3 | Left Inferior fronto-occipital fasciculus | AD | 0.766 |
| 4 | Right Sagittal stratum | FA | 0.756 |
| 5 | Left Anterior limb of internal capsule | AD | 0.750 |
| 6 | Bilateral Sagittal stratum | FA | 0.729 |
| 7 | Left Uncinate fasciculus | FA | 0.727 |
| 8 | Bilateral Superior fronto-occipital fasciculus | FA | 0.718 |
| 9 | Left Posterior limb of internal capsule | FA | 0.715 |
| 10 | Left Superior fronto-occipital fasciculus | FA | 0.703 |

##

## Supplementary Table 5. Top 20 features of medication classification models. (A) Classification model of medicated OCD from unmedicated OCD in adult samples (medicated OCD: N = 261, unmedicated OCD: N = 429). (B) Classification model of medicated OCD from unmedicated OCD in pediatric samples (medicated OCD: N = 70, unmedicated OCD: N = 105)

**(A) Classification model of medicated OCD from unmedicated OCD in adult samples** (medicated OCD: N = 261, unmedicated OCD: N = 429)

| **Rank** | **Feature** | **DTI Metric** | **Weight** |
| --- | --- | --- | --- |
| 1 | Corpus Callosum | AD | 1.000 |
| 2 | Left Posterior limb of internal capsule | FA | 0.726 |
| 3 | Average FA | FA | 0.476 |
| 4 | Right Inferior fronto-occipital fasciculus | AD | 0.308 |
| 5 | Bilateral Fornix and Stria terminalis | MD | 0.308 |
| 6 | Bilateral Cingulum (hippocampus) | RD | 0.267 |
| 7 | Genu of corpus callosum | FA | 0.261 |
| 8 | Average RD | RD | 0.253 |
| 9 | Genu of corpus callosum | AD | 0.228 |
| 10 | Right Cingulum (hippocampus) | RD | 0.222 |

**(B) Classification of medicated OCD from unmedicated OCD in pediatric samples** (medicated OCD: N = 70, unmedicated OCD: N = 105)

| **Rank** | **Feature** | **DTI Metric** | **Weight** |
| --- | --- | --- | --- |
| 1 | Bilateral Fornix (cres)/Stria terminalis | AD | 1.000 |
| 2 | Left Anterior corona radiata | RD | 0.971 |
| 3 | Bilateral Cingulum (cingulate gyrus) | AD | 0.854 |
| 4 | Bilateral Uncinate fasciculus | AD | 0.833 |
| 5 | Right Cingulum (cingulate gyrus) | AD | 0.800 |
| 6 | Bilateral Cingulum (hippocampus) | AD | 0.791 |
| 7 | Left Internal capsule | AD | 0.790 |
| 8 | Bilateral Posterior limb of internal capsule | FA | 0.722 |
| 9 | Right Anterior limb of internal capsule | FA | 0.692 |
| 10 | Left Anterior limb of internal capsule | AD | 0.685 |

##

## Supplementary Table 6. Top 20 features to classify diagnosis derived from LIME. (A) Classification of OCD from HC in adult samples (OCD: N = 690, HC: N = 646), (B) Classification of OCD from HC in pediatric samples (OCD: N = 175, HC: N = 142), (C) Classification of unmedicated OCD (N = 429) and HC (N = 646) in adult samples.

## (A) Classification of OCD from HC in adult samples (OCD: N = 690, HC: N = 646)

|  | **Features positively associated  with adult OCD** | | | **Features negatively associated  with adult OCD** | | |
| --- | --- | --- | --- | --- | --- | --- |
| **Rank** | **Feature** | **DTI**  **Index** | **Weight** | **Feature** | **DTI**  **Index** | **Weight** |
| 1 | Bilateral Corticospinal tracts | RD | 0.091 | Corpus callosum | AD | 0.039 |
| 2 | Bilateral Posterior limb of internal capsule | FA | 0.069 | Corpus callosum | MD | 0.036 |
| 3 | Corpus callosum | FA | 0.050 | Bilateral Superior corona radiata | FA | 0.036 |
| 4 | Bilateral Internal capsule | FA | 0.045 | Right Fornix/Stria terminalis | RD | 0.035 |
| 5 | Bilateral Corona radiata | FA | 0.044 | Right Corona radiata | AD | 0.035 |
| 6 | Bilateral Fornix/Stria terminalis | MD | 0.038 | Bilateral Internal capsule | AD | 0.033 |
| 7 | Right Posterior limb of internal capsule | AD | 0.035 | Bilateral Uncinate fasciculus | RD | 0.033 |
| 8 | Bilateral Internal capsule | RD | 0.033 | Bilateral Fornix/Stria terminalis | AD | 0.032 |
| 9 | Bilateral Anterior corona radiata | RD | 0.030 | Bilateral Superior corona radiata | FA | 0.032 |
| 10 | Bilateral Superior corona radiata | AD | 0.029 | Bilateral Anterior corona radiata | AD | 0.031 |
| The interpretation model accounts for 45.69% of the variance | | | | | | |

**(B) Classification of OCD from HC in pediatric samples**  (OCD: N = 175, HC: N = 142)

|  | **Features positively associated  with pediatric OCD** | | | **Features negatively associated  with pediatric OCD** | | |
| --- | --- | --- | --- | --- | --- | --- |
| **Rank** | **Feature** | **DTI**  **Index** | **Weight** | **Feature** | **DTI**  **Index** | **Weight** |
| 1 | Left Posterior thalamic radiation | RD | 0.350 | Left Posterior thalamic radiation | AD | 0.370 |
| 2 | Right Retrolenticular part of the internal capsule | MD | 0.330 | Right External capsule | AD | 0.310 |
| 3 | Left Fornix/Stria terminalis | AD | 0.330 | Bilateral Superior corona radiata | FA | 0.280 |
| 4 | Right Posterior thalamic radiation | AD | 0.310 | Left Cingulum cingulate gyrus part | RD | 0.270 |
| 5 | Left Uncinate fasciculus | RD | 0.30 | Right Anterior limb of internal capsule | RD | 0.260 |
| 6 | Bilateral Inferior fronto-occipital fasciculus | RD | 0.280 | Left Anterior corona radiata | RD | 0.250 |
| 7 | Right External capsule | MD | 0.270 | Bilateral Inferior fronto-occipital fasciculus | FA | 0.250 |
| 8 | Right External capsule | RD | 0.260 | Right Posterior thalamic radiation | RD | 0.250 |
| 9 | Right Cingulum cingulate gyrus part | RD | 0.260 | Left Sagittal stratum | MD | 0.240 |
| 10 | Corpus callosum | FA | 0.260 | Left Retro Lenticular part of internal capsule | MD | 0.240 |
| The interpretation model accounts for 47.12% of the variance. | | | | | | |

## (C) Classification of unmedicated OCD (N = 429) and HC (N = 646) in adult samples

|  | **Features positively associated  with unmedicated OCD** | | | **Features negatively associated  with unmedicated OCD** | | |
| --- | --- | --- | --- | --- | --- | --- |
| **Rank** | **Feature** | **DTI**  **Index** | **Weight** | **Feature** | **DTI**  **Index** | **Weight** |
| 1 | Bilateral Posterior limb of internal capsule | FA | 0.200 | Bilateral Cingulum cingulate gyrus part | MD | 0.130 |
| 2 | Bilateral Cingulum hippocampal part | MD | 0.170 | Right Cingulum hippocampal part | RD | 0.130 |
| 3 | Bilateral Posterior limb of internal capsule | MD | 0.120 | Left Posterior thalamic radiation | RD | 0.088 |
| 4 | Left Posterior thalamic radiation | MD | 0.100 | Right Fornix/Stria terminalis | RD | 0.083 |
| 5 | Bilateral Corona radiata | FA | 0.093 | Bilateral Cingulum hippocampal part | AD | 0.080 |
| 6 | Bilateral Posterior limb of internal capsule | RD | 0.090 | Bilateral Posterior corona radiata | MD | 0.075 |
| 7 | Bilateral Corona radiata | AD | 0.085 | Bilateral Anterior corona radiata | AD | 0.071 |
| 8 | Right Posterior limb of internal capsule | RD | 0.080 | Bilateral Fornix/Stria terminalis | MD | 0.068 |
| 9 | Right Cingulum cingulate gyrus part | MD | 0.072 | Bilateral Superior corona radiata | FA | 0.064 |
| 10 | Right Cingulum hippocampal part | MD | 0.070 | Corpus callosum | MD | 0.056 |
| Note that the target class is unmedicated OCD.  The interpretation model accounts for 63.8% of the variance. | | | | | | |

## Supplementary Table 7. Top 20 features to differentiate between adult unmedicated OCD (N = 429) and adult medicated OCD (N = 261) derived from LIME.

|  | **Features positively associated  with OCD medication** | | | **Features negatively associated  with OCD medication** | | |
| --- | --- | --- | --- | --- | --- | --- |
| **Rank** | **Feature** | **DTI**  **Index** | **Weight** | **Feature** | **DTI**  **Index** | **Weight** |
| 1 | Bilateral Posterior corona radiata | RD | 0.005 | Corpus callosum | AD | 0.008 |
| 2 | Bilateral Posterior limb of internal capsule | RD | 0.004 | Average RD | | 0.007 |
| 3 | Bilateral Superior corona radiata | RD | 0.007 | Left Posterior limb of internal capsule | FA | 0.005 |
| 4 | Right Inferior fronto-occipital fasciculus | AD | 0.002 | Corpus callosum | MD | 0.004 |
| 5 | Right Posterior corona radiata | MD | 0.002 | Bilateral Anterior corona radiata | MD | 0.003 |
| 6 | Splenium of corpus callosum | MD | 0.002 | Right Anterior limb of internal capsule | MD | 0.003 |
| 7 | Splenium of corpus callosum | AD | 0.002 | Right Internal capsule | RD | 0.003 |
| 8 | Bilateral Superior corona radiata | AD | 0.002 | Bilateral Superior corona radiata | FA | 0.002 |
| 9 | Splenium of corpus callosum | RD | 0.002 | Bilateral Corticospinal tract | RD | 0.002 |
| 10 | Left Posterior thalamic radiation | MD | 0.002 | Right Fornix/Stria terminalis | FA | 0.002 |
| Note that the target class is medicated OCD.  The interpretation model accounts for 59.8% of the variance. | | | | | | |

##

## Supplementary Table 8. Performance of adult-trained models in pediatric OCD prediction – mean with 95% confidence interval.

|  | **OCD vs. HC** | |
| --- | --- | --- |
|  | **Trained, validated  on adult samples** | **Tested  on pediatric samples** |
| ROC-AUC | 63.91 (52.4-72.3) | 55.81 (49.5-62.2) |
| Accuracy (%) | 64.81 (57.1-75.0) | 57.41 (51.7-63.4) |
| Sensitivity (%) | 65.2 (33.2-91.4) | 52.57 (19.4-80) |
| Specificity (%) | 64.47 (35.8-91.6) | 63.38 (34.5-92.3) |

As a secondary analysis, we assessed differences in matter microstructure between adult OCD and pediatric OCD participants. We tested the generalizability of the model trained on the adult samples (N=1,336; OCD: 690, HC: 640) to the pediatric samples (N=317; OCD: 175, HC: 142). The model trained on adult samples showed moderately accurate performance in classifying adult OCD participants from HCs (ROC-AUC = 63.91, 95% CI = 52.4-72.3). This model provided lower performance when applied to pediatric samples (ROC-AUC =55.81, 95% CI =49.5-62.2)**.**

| **Rank** | **Feature** | **DTI** | **Weight** |
| --- | --- | --- | --- |
|  |  | **Index** |  |
| 1 | R Inferior fronto-occipital fasciculus | FA | 1.000 |
| 2 | L Posterior thalamic radiation | FA | 0.872 |
| 3 | L Posterior limb of internal capsule | AD | 0.826 |
| 4 | L Posterior corona radiata | FA | 0.750 |
| 5 | L Uncinate fasciculus | FA | 0.377 |
| 6 | R Superior longitudinal fasciculus | RD | 0.316 |

## Supplementary Figure 1. Leave-one-site-out cross-validation.

##
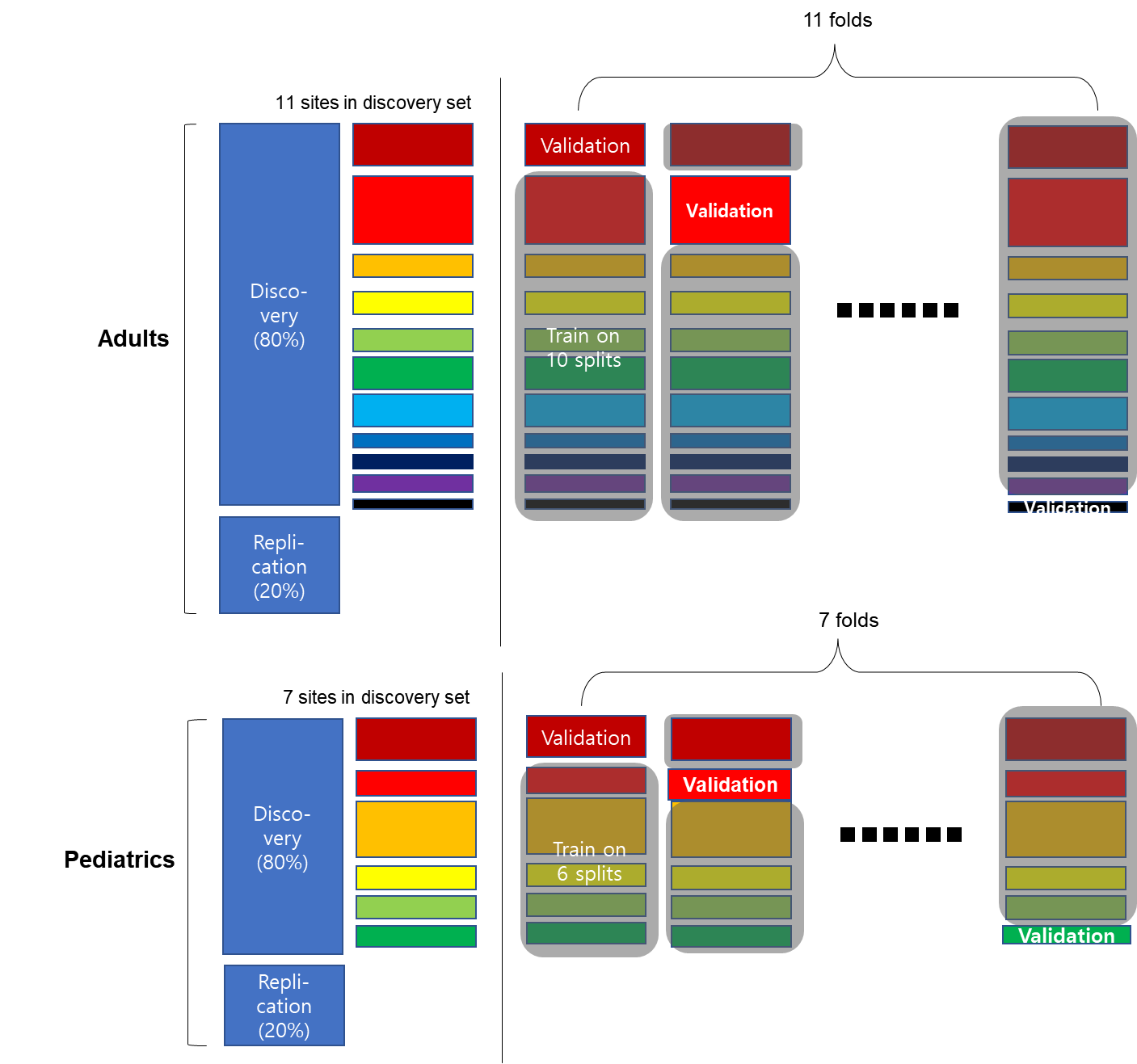


##

## Supplementary Figure 2. Feature importance plot of the diagnosis and medication models in adult (Left) and pediatric (Right) samples. (A), relative importance plot of OCD vs. HC model. (B), the relative importance of unmedicated OCD vs. HC model. (C), relative importance plot of medicated OCD vs. unmedicated OCD model. The top 10 features are represented.


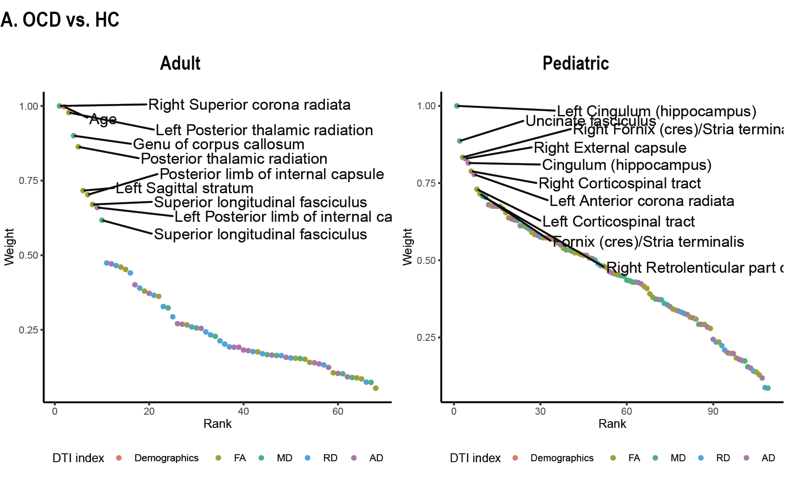


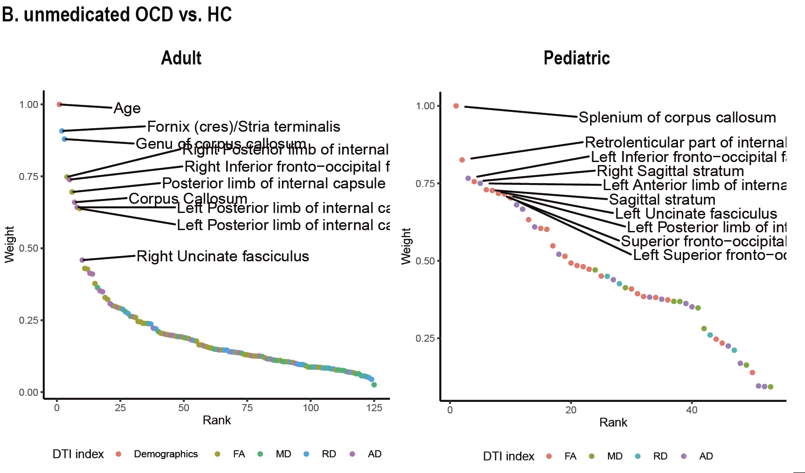


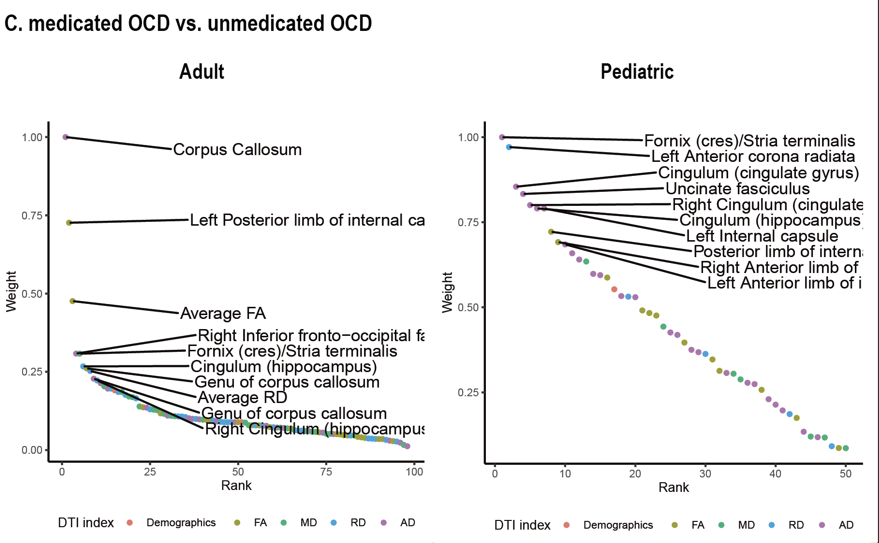

Supplement: Supplementary file 1 — Supplementral material [file 41380_2023_2392_MOESM1_ESM.docx]
